# Machine learning analysis of CO_2_ and methane adsorption in tight reservoir rocks

**DOI:** 10.1038/s41598-025-10010-4

**Published:** 2025-07-08

**Authors:** Mehdi Maleki, Mohammad Rasool Dehghani, Moein Kafi, Ali Akbari, Yousef Kazemzadeh, Ali Ranjbar

**Affiliations:** Department of Petroleum Engineering, Faculty of Petroleum, Gas, and Petrochemical Engineering, Persian Gulf University, Bushehr, Iran

**Keywords:** Underground gas storage, CO_2_, CH_4_, Gas adsorption, Life cycle assessment, Thermodynamic parameter analysis, Greenhouse gases, Engineering, Chemical engineering

## Abstract

Greenhouse gases, particularly CO_2_ and CH_4_, are key contributors to climate change and global warming. Consequently, effective management and reduction of these emissions, especially in subsurface storage applications, are crucial. Adsorption presents a promising strategy for mitigating CO_2_ and CH_4_ emissions in the energy sector, particularly in the storage and utilization of fossil fuel resources, thereby minimizing the environmental impact of their extraction and consumption. In this study, the adsorption behavior of CO_2_ and CH_4_ in tight reservoirs is examined using experimental data and advanced machine learning (ML) techniques. The dataset incorporates key variables such as temperature, pressure, rock type, total organic carbon (TOC), moisture content, and the CO_2_ fraction in the injected gas. Various ML models were employed to predict gas adsorption capacity, with CatBoost and Extra Trees demonstrating high predictive performance. The CatBoost model achieved superior results, with R² values of 0.9989 for CO₂ and 0.9965 for CH₄, along with low RMSE and MAE values, indicating strong stability and accuracy across all metrics. Sensitivity analysis identified pressure as the most influential factor, followed by TOC and CO_2_ percentage, while temperature had a restrictive effect on adsorption. Secondary variables, such as rock type and moisture content, also contributed, though to a lesser extent. Graphical analyses further validated the high accuracy of the ML models, particularly CatBoost and Extra Trees. The findings underscore the effectiveness of ML approaches and optimized hyperparameter tuning in enhancing the prediction of gas adsorption capacity, thereby improving the design of gas injection and storage processes. This research provides valuable insights for optimizing gas composition and operational parameters in storage applications, serving as a foundation for future studies in gas sequestration and reservoir engineering.

## Introduction

The adsorption process is a critical component in material purification and separation, offering a cost-effective and efficient solution for addressing environmental challenges^1^. Gas adsorption, in particular, plays a significant role in applications such as methane (CH_4_) and carbon dioxide (CO_2_) storage in geological formations^2^. During this process, gas molecules adhere to the pore walls of porous materials in reservoirs through physical forces (e.g., van der Waals forces) or chemical interactions (e.g., covalent bonds). Key factors influencing adsorption include pressure, temperature, pore size and structure, gas composition, and the chemical and physical properties of the reservoir rock. At low pressures, monolayer adsorption occurs, while high pressures may lead to multilayer adsorption. Organic-rich rocks like coal and shale exhibit high adsorption capacities for CH_4_ and CO_2_, making them crucial for gas storage, unconventional gas production, and greenhouse gas mitigation^3–7^.

Coalbed methane (CBM) reservoirs and shale formations are recognized as promising candidates for greenhouse gas storage^8–10^. In CBM reservoirs, storage predominantly occurs through adsorption, whereas in shale formations, both adsorbed and free gas phases contribute to their storage capacity. Advanced recovery techniques, such as enhanced CBM recovery (ECBM) and enhanced shale gas recovery (ESGR), utilize CO_2_ injection to replace CH_4_, enhancing methane production while simultaneously storing CO_2_. CBM reservoirs, characterized by their porous structure and high organic content, efficiently adsorb methane (CH_4_) through physical adsorption onto coal pore surfaces. This process is directly influenced by reservoir pressure, with higher pressures resulting in increased adsorption capacity. Unlike conventional reservoirs, where gas is stored as compressed free gas in void spaces, gas in CBM reservoirs binds to coal surfaces via van der Waals forces, making them ideal for natural gas production and CO_2_ storage. CO_2_ injection not only enhances methane production but also contributes to greenhouse gas mitigation^11–17^.

In shale formations, gas storage involves a combination of adsorption onto pore surfaces and free gas storage within pore spaces. Shale’s small pore sizes and unique mineral and organic compositions enable strong interactions with CO_2_, resulting in remarkable adsorption capacities. Factors such as TOC, moisture content, mineral composition, reservoir temperature, and pressure significantly influence adsorption capacity. These properties position shales as a viable option for greenhouse gas storage and unconventional gas production^18–23^.

The adsorption and desorption processes in CBM and shale reservoirs are governed by their porous structures and organic content, which facilitate significant CO_2_ uptake through strong chemical and physical interactions with the rock matrix. In shale formations, adsorption mechanisms include monolayer and multilayer adsorption, influenced by reservoir pressure. Compared to nonpolar CH_4_ molecules, CO_2_ forms stronger bonds with organic functional groups, enabling preferential adsorption^24–27^. Parameters such as TOC, mineral composition, and moisture content play critical roles; higher TOC correlates with greater adsorption capacity, while moisture acts as a competing agent, reducing efficiency^28,29^. Gas storage occurs either as compressed free gas in pore spaces or adsorbed onto pore walls, and these mechanisms are vital for applications like greenhouse gas mitigation, energy storage, and unconventional gas production^30–32^.

Recent advancements in machine learning (ML) have provided robust tools for predicting gas adsorption capacity. Studies have employed algorithms such as artificial neural networks (ANN), least squares support vector machines (LSSVM), and other ML methods to model the adsorption behavior of CO_2_ and CH_4_. These models leverage experimental datasets to identify key parameters such as TOC, moisture content, and thermodynamic conditions, achieving superior accuracy compared to traditional isotherm models. The integration of ML techniques offers precise and efficient predictions of gas adsorption behavior under varying reservoir conditions.

In 2024, Tavakolian et al.^1^ evaluated ML methods for modeling CH_4_ and CO_2_ adsorption capacities in tight reservoirs like shale and coal seams, using 3,804 gas adsorption data points with shallow and deep learning models. Their analysis revealed that the Random Forest (RF) algorithm outperformed others, achieving high accuracy in predicting CH_4_ (MAE = 0.0864, RMSE = 0.1520) and CO_2_ (MAE = 0.0529, RMSE = 0.2308) adsorption capacities. Sensitivity analysis highlighted the alignment of ML models with geological and reservoir engineering principles, underscoring their potential for laboratory and simulation applications. In 2024, Zhou et al.^33^ developed a Gaussian Process Regression (GPR) model to predict methane adsorption capacity in shale formations. Using experimental data from the Longmaxi formation in the Sichuan Basin, five key variables—TOC, clay minerals, temperature, pressure, and moisture—were identified as significant. The GPR model outperformed the Extreme Gradient Boosting (XGBoost) model, achieving a relative prediction error below 3%. Sensitivity analysis indicated that TOC was the most influential factor, while clay minerals influenced adsorption through interactions with other variables.

In another study, in 2024, Wang et al.^34^ introduced an innovative approach combining molecular simulation, the lattice Boltzmann method, and ML to predict CO_2_-CH_4_ competitive adsorption in large-scale porous shale environments. By training an ANN on molecular simulation data, this method overcame computational limitations and incorporated variables such as shale mineral type and CO_2_ mole fractions. This approach provides a new foundation for modeling adsorption behavior in porous media, facilitating CO_2_ sequestration and enhanced CH_4_ recovery. In 2024, according to the study conducted by Alqahtani et al.^35^ the objective of this research was to develop a data-driven framework for predicting the adsorption capacity of methane (CH_4_) and CO_2_ in unconventional reservoirs such as shale and coal. The study utilized three intelligent models, including General Regression Neural Network (GRNN), Radial Basis Function Neural Network (RBFNN), and CatBoost, which were trained and tested with over 3,800 real data points related to CH_4_ and CO_2_ adsorption. To improve model performance, the structure and control parameters of RBFNN and CatBoost were automatically optimized using the Grey Wolf Optimization (GWO) method. The results indicated that the CatBoost-GWO combined model provided the most accurate results with RMSE values of 0.1229 and 0.0681 and R^2^ values of 0.9993 and 0.9970 for CO_2_ and CH_4_ adsorption, respectively. Additionally, the model effectively maintained the physical adsorption trends compared to operational parameters and demonstrated superior performance compared to recent ML methods.

In 2023, Alanazi et al.^36^ proposed an ML framework for predicting CO_2_ adsorption capacity in coal seams using a dataset of 1,064 experimental data points. Among various ML techniques, RF demonstrated the highest accuracy, particularly for CO_2_ adsorption at higher pressures. This framework reduces reliance on extensive experiments and complex mathematical models. In 2023, Kalam et al.^37^ employed Gradient Boosting Regression to predict hydrogen adsorption on kerogen shale for underground storage. This model achieved high accuracy, with a determination coefficient of 99.6% on training data and 94.6% on test data, demonstrating the significance of kerogen type on hydrogen adsorption. This approach significantly reduces the time required for laboratory experiments and molecular simulations.

In 2022, Amar et al.^38^ utilized Genetic Expression Programming (GEP) to model CH_4_ adsorption in shale gas formations. Their results revealed that CH_4_ adsorption is strongly influenced by humidity, pressure, TOC, and temperature. The GEP model exhibited a high correlation coefficient (0.9837), providing user-friendly equations for estimating adsorption capacity. In 2020, Meng et al.^39^ and Wang et al.^40^ explored ML models for predicting methane adsorption in shale and gas content in shale reservoirs. Meng et al. evaluated classical isothermal and pressure-temperature integrated models alongside ML methods like ANN, RF, SVM, and XGBoost, with XGBoost showing superior performance by addressing limitations of isothermal conditions and accurately predicting beyond experimental ranges. Similarly, Wang et al. used over 700 data points to compare models such as MLR, SVM, RF, and ANN, identifying RF as the most reliable for predicting Langmuir parameters with high accuracy (R^2^ = 0.84–0.87). Both studies emphasized the potential of ML for improving accuracy, reducing costs, and optimizing shale gas production and reservoir simulations.

**Table 1:** Overview of previous research.

No.	Author(s)	Research Objective	Method Used	Theoretical Results	Numerical Results
1	Tavakolian et al. (2024)^1^	Prediction of CH_4_ and CO_2_ Adsorption Capacity in Tight Reservoirs	Utilization of ML Methods Including RF and Hyperparameter Tuning with Optuna	The RF method demonstrated the best performance for predicting adsorption capacity.	CH_4_: MAE = 0.0864, RMSE = 0.1520; CO_2_: MAE = 0.0529, RMSE = 0.2308
2	Zhou et al. (2024)^33^	Modeling of CH_4_ Adsorption in Shale Using GPR	Development of GPR Model and Comparison with XGBoost	GPR was the most accurate method; TOC was the most influential variable.	Reduction of prediction error to less than 3%
3	Wang et al. (2024)^34^	Prediction of Competitive CO_2_-CH_4_ Adsorption in Shale Porous Media	Integration of Molecular Simulation, Boltzmann Network, and ANN	Computational limitations were addressed; the impact of mineral type was examined.	-
4	Alanazi et al. (2023)^36^	Prediction of CO_2_ Adsorption Capacity in Coal	ML Models Including RF, ANN, and ANFIS	RF provided the most accurate predictions.	Low RMSE and AAPE at high pressures
5	Amar et al. (2022)^38^	Modeling of CH_4_ Adsorption Capacity in Shale	Application of GEP and GMDH	GEP was more precise with mathematical relationships.	R^2^ = 0.9837; Moisture has a greater impact than TOC
6	Kalam et al. (2023)^37^	Prediction of Hydrogen Adsorption in Shale	Use of Gradient Boosted Regression	Data-driven models were more accurate and faster.	Coefficient of determination: 99.6% (training), 94.6% (testing)
7	Meng et al. (2020)^39^	Prediction of CH_4_ Adsorption for Shale Production Planning	Comparison of ML with Classical Models	XGBoost showed the best performance.	Accurate predictability for TOC, temperature, and moisture
8	Alqahtani et al. (2024)^35^	Prediction of CH_4_ and CO_2_ Adsorption in Shale and Coal Reservoirs	Optimized GRNN, RBFNN, and CatBoost Models	CatBoost-GWO was the most accurate model.	CO_2_: RMSE = 0.1229; CH_4_: RMSE = 0.0681

Table 1 summarizes the research background, emphasizing the challenges of predicting gas adsorption in unconventional reservoirs for natural gas and CO_2_ production and storage. ML methods have emerged as faster, more accurate alternatives to traditional models and costly experiments. Studies show that optimized ML models, such as XGBoost and CatBoost-GWO, achieve high accuracy (R^2^ > 0.99) and low error rates (RMSE < 0.1), enhancing predictions and enabling large-scale simulations. These models address the limitations of classical methods, reduce computational costs, and support reservoir design, reserve assessment, and gas recovery optimization. ML-based workflows also predict anomalous CO_2_ adsorption under high-pressure conditions and enable accurate estimation of adsorption capacity based on CO_2_ injection percentage, rock type, and thermodynamic conditions. The research demonstrates the reliability and practicality of ML techniques in advancing gas adsorption predictions and reservoir management.

Recent advancements in data-driven modeling have significantly enhanced the understanding of complex subsurface phenomena, particularly in tight reservoirs where conventional modeling techniques may fall short. This study presents a novel, data-centric approach to predicting CO_2_ and CH_4_ adsorption capacities using a comprehensive experimental dataset under various thermodynamic conditions. By integrating advanced machine learning algorithms. This research not only benchmarks model performance but also reveals critical insights into the governing parameters of gas adsorption. The application of these ensemble learning methods provides a robust framework for capturing nonlinear interactions, thereby offering a more accurate and generalizable prediction of gas behavior in tight formations.

This research focuses on modeling gas adsorption using machine learning in unconventional hydrocarbon reservoirs such as coal and shale using an experimental dataset published by Tavakolian et al.^1^. The authors provide a comprehensive dataset containing methane and carbon dioxide adsorption data from a variety of thermodynamic and geologic environments throughout the world. The authors have developed many machine learning models to estimate gas adsorption capacity based on pressure, temperature, rock type, TOC, moisture content and gas composition. This research intends on analyzing the unexplored machine learning techniques with one unified modeling framework. In this way, we can also compare the prediction quality versus the best-known machine learning prediction models outlined in previous works. Additionally, by performing a structured analysis of a variety of new machine learning approaches, we will provide an unbiased representation of the capabilities of current methods being used for predicting gas adsorption using current experimental data.

## Data collection and specific description

The experimental dataset used in this study was compiled from the published work of Tavakolian et al.^1^. The dataset includes adsorption measurements of CH_4_ and CO_2_ in shale and coal reservoirs under a wide range of pressures, temperatures, and reservoir properties. Detailed descriptions of the experimental setup, data distribution, and statistical characteristics have been thoroughly reported in the original study and are therefore not repeated here. In the present work, the dataset is directly utilized as input for machine learning model development and comparative analysis.

### Machine learning model

Due to the nonlinear and multivariate characteristics of gas sorption processes, as well as the heterogeneity of the experimental dataset, we chose ensemble-based machine learning techniques during this study for their capacity to effectively model complicated interactions between variables and provide less variance in comparison to single models when predicting outcomes from tabular experimental datasets.

Random Forest is a well-known ensemble algorithm that is based on bootstrap aggregation, retains strong performance, and is robust to overfitting when modeling nonlinear relationships. The Extra Trees ensemble method has been selected due to its similarity to the Random Forest method with the introduction of additional randomness when constructing trees. The additional randomization of the Extra Trees method will allow for an evaluation of whether the increased amount of randomness will improve generalization for the dataset in question.

AdaBoost is an example of a boosting type of ensemble technique that focuses on improving prediction accuracy through iterative treatment of samples that are difficult to predict based on their resampling through adaptive reweighting of instances. CatBoost is an example of a more recent variation of the Gradient Boosting Algorithm that performs particularly well with respect to complex nonlinear relationships and the structured nature of tabular format datasets.

Choosing these models allows for a standardized comparison of different ensemble learning techniques like bagging, randomization-based ensembles, and boosting techniques, all within a unified modeling framework. Simpler linear regression models were not considered, as they are generally insufficient to represent the highly nonlinear adsorption behavior observed in experimental adsorption datasets.

#### Random forest

**Fig. 1:**
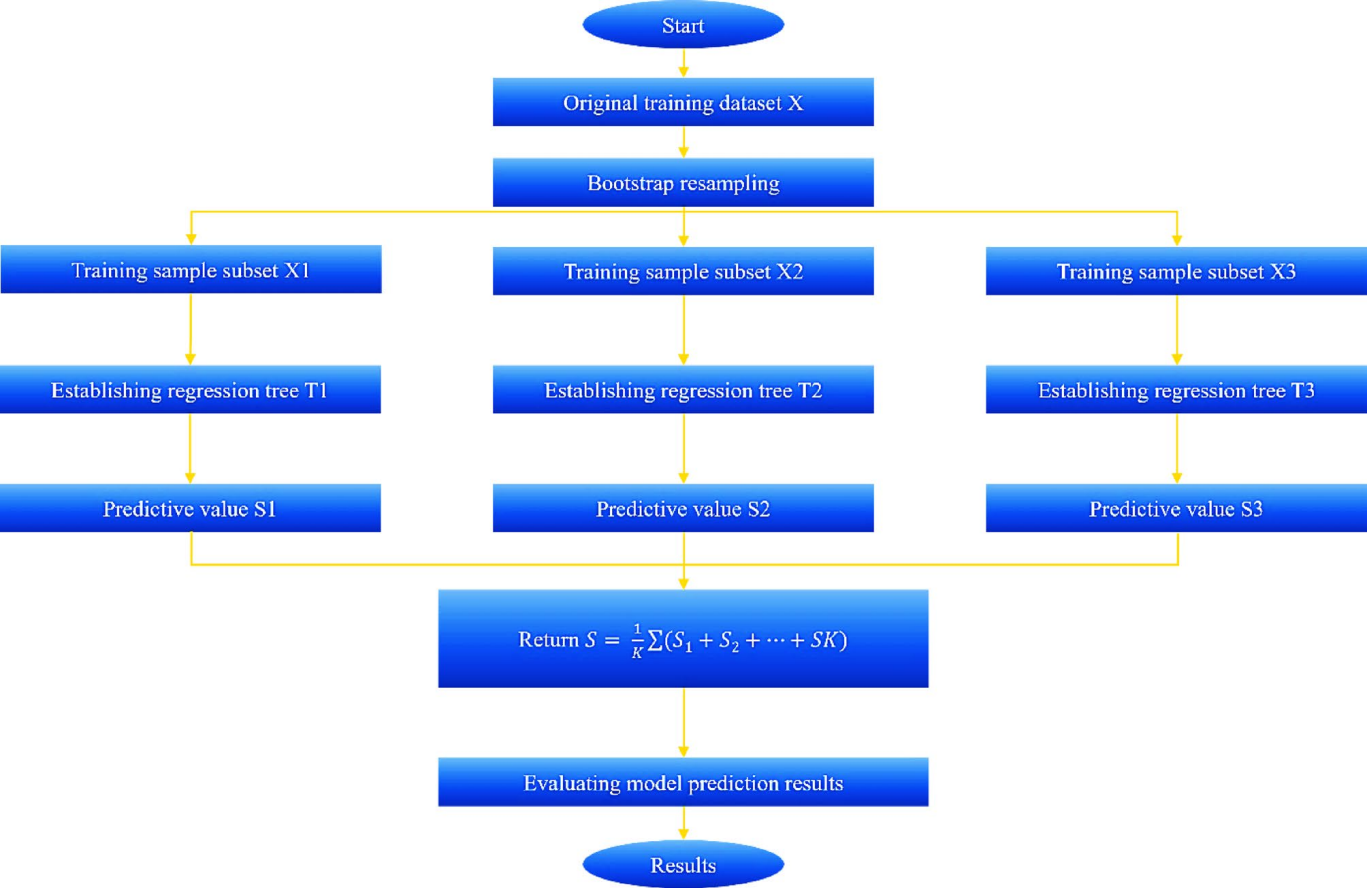
RF Flowchart.

Due to its non-parametric nature and ability to efficiently handle large datasets, the RF algorithm can achieve high performance in studies of this type. RF is an ensemble model of decision trees (DTs), with each DT constructed using the Classification and Regression Trees (CART) method^41^. By utilizing a random subset of the training data and random features at each split, RF reduces variance and provides better generalization^42^. This algorithm combines the interpretability of DT with the robustness of ensemble learning, resulting in higher predictive power and a reduced risk of overfitting. Random Forest Regression (RFR) is an advanced version of the Decision Tree Regression (DTR) algorithm, leveraging these advantages to enhance performance^43^. A flowchart of this model is shown in Fig. 1.

In this study, RF was implemented using the Scikit-learn library in Python and relied on the Bootstrap Aggregation (Bagging) method to independently construct DTs, which reduces the variance errors associated with individual models^44^. The final regression prediction is obtained by averaging all the predicted values from each tree, thereby enhancing the accuracy and robustness of the model^45^. The RFR algorithm performed several key steps^46^:

1. **Bootstrap Sampling**: The training set was sampled k times using the bootstrap method, creating k subsets of the training data with equal sizes.
2. **Feature Selection and Tree Construction**: For data with M features, a random subset of m (M > m) features was selected from all M features to be used as candidate feature subsets for a node. The feature impurity index was then used to identify the best node and branch, and k DTR models were constructed.
3. **Final Prediction**: The average of the k predictions was calculated to provide the final regression result.

#### Categorical boosting

**Fig. 2:**
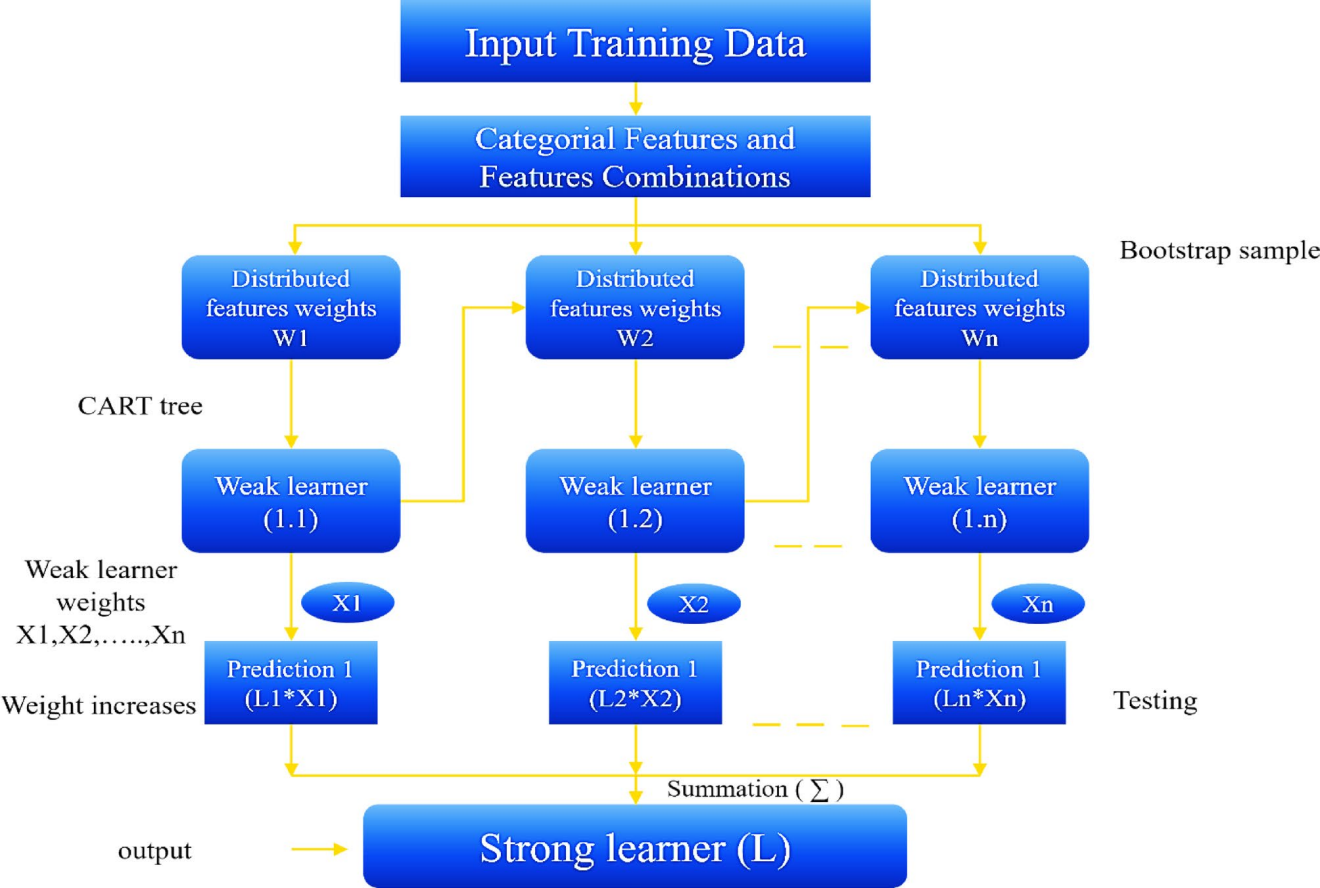
CatBoost Flowchart.

Advanced ML algorithms, such as CatBoost, have been developed to address the limitations of individual models. CatBoost is a member of the Gradient-Boosted Decision Trees (GBDT) family and is primarily recognized for its exceptional capabilities in processing categorical features. One of the key features of CatBoost is that it does not require extensive preprocessing of categorical data, which is often a time-consuming task in other gradient boosting frameworks. CatBoost operates differently; it utilizes advanced methods such as Ordered Boosting and Target Encoding to handle overfitting (Fig. 2)^47–49^.

Compared to other gradient boosting techniques that typically require categorical variables to be converted into numerical data, CatBoost can directly handle categorical features, significantly reducing the amount of preprocessing needed. By processing categorical data directly, CatBoost can leverage key information more effectively, making the model more efficient. As part of the GBDT framework, CatBoost constructs a series of DTs sequentially, with each tree aiming to capture the residual errors of the previous trees. Weights are adjusted based on the prediction errors of the training samples, adapting the model to more challenging samples.

CatBoost also employs unique strategies for performance optimization. For example, Ordered Boosting, where trees are ordered based on combinations of a feature rather than random or sequential orders, improves the model’s accuracy by focusing on more informative features. Additionally, CatBoost uses Oblivious Trees^50,51^, which allow for parallel computation during the training process, resulting in time savings and improved performance. Finally, CatBoost organizes the training samples in a fixed order and gradually increases the number of training samples for each model. This systematic and gradual learning process offers advantages over building a model at each iteration, as it helps progressively improve performance^52^.

#### Adaptive boosting

**Fig. 3:**
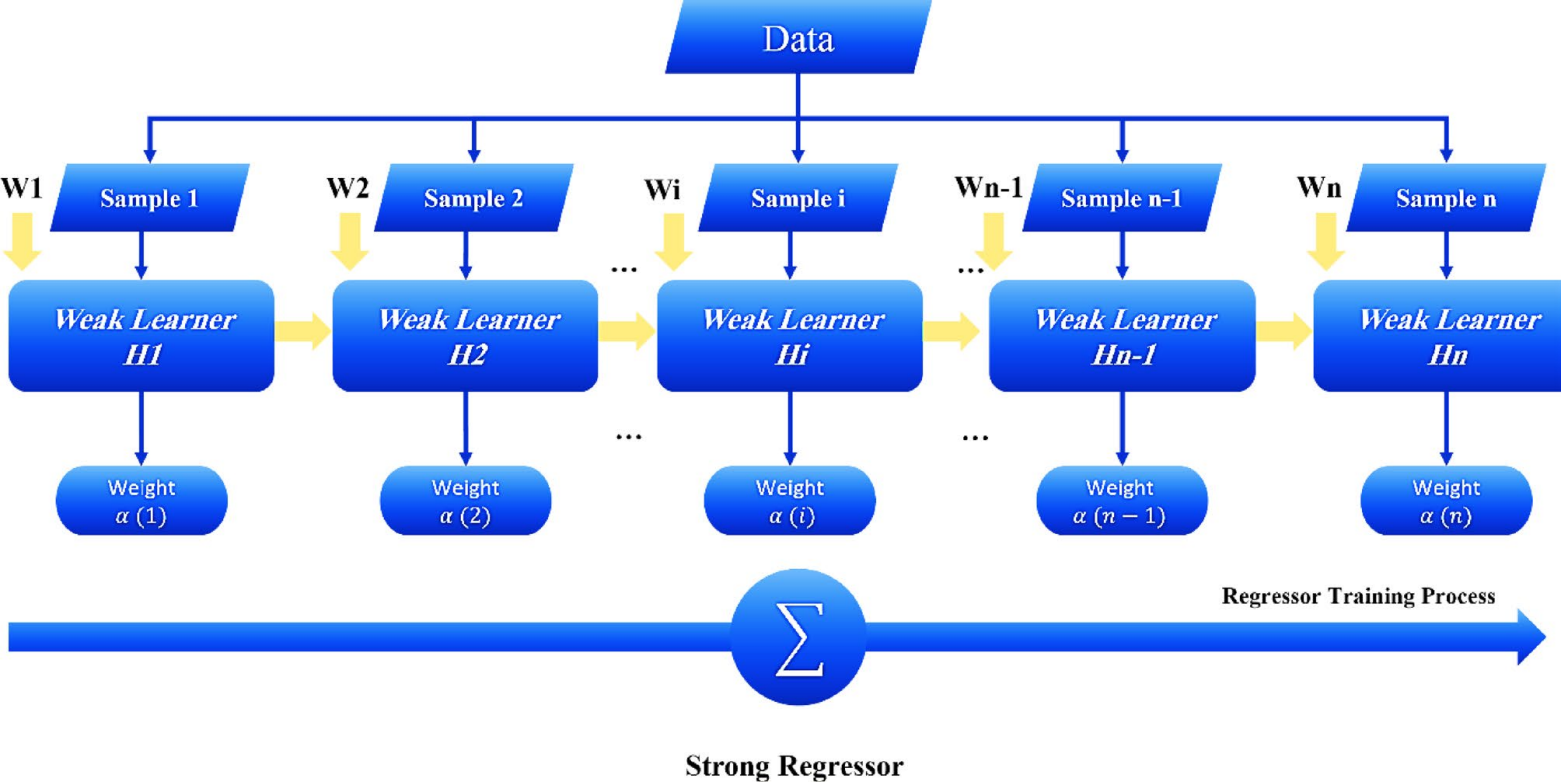
AdaBoost flowchart.

As shown in Fig. 3, Boosting is a ML technique used to combine multiple weak models, such that the resulting model has better predictive accuracy than any individual model. AdaBoost, one of the most well-known types of boosting, is a sequential ensemble learning method that gradually improves model performance by correcting the weights of misclassified data points in previous models^53–55^.

In this algorithm, a weak learner, often a DT, is first trained on the original dataset. In each iteration, the algorithm adjusts the weights of the training data and places more emphasis on the data points that were misclassified in previous iterations. This process is cyclical, with predictions being continuously improved, and each subsequent model leading to a more accurate result.

At each stage, AdaBoost increases the weight of misclassified samples to ensure that the next weak learner focuses more on them. Ultimately, all the weak learners are combined, and the final model is created, with each learner being weighted according to its performance.

One important aspect of AdaBoost is its ability to combine weak learners, which can be applied with techniques such as Support Vector Regression (SVR) or DTR. AdaBoost has proven to perform well in both classification and regression tasks and typically outperforms other ensemble methods in terms of accuracy.

However, this algorithm is not without limitations. Some weaknesses of AdaBoost include its sensitivity to outliers and noisy data, as incorrect samples receive higher weights. Additionally, due to the number of iterations required for training, the algorithm is computationally expensive and may lead to overfitting if the weak learners are too complex or the dataset is too small^56–58^.

#### Extra trees regressor

Extra Trees Regressor (ETR) is an ensemble learning method that operates by creating a large number of DTs independently. In this method, at each node, a feature and the branching value are selected randomly^59,60^. Similar to the RF algorithm, which is also based on an ensemble of DTs, ETR differs in its training and branching approach. Specifically, RF uses bootstrap sampling (randomly creating subsets of data with replacement) and finds the best branches using criteria such as Gini impurity or mean squared error (MSE). In contrast, the ETR algorithm is trained on the entire dataset and selects features and branching values randomly at each node. This additional randomness in the branching phase often results in better performance for ETR, especially when overfitting is a concern^61^.

To separate the nodes, ETR randomly selects binary branching values, while RF determines a set of candidates branching values for each feature and selects the best one based on optimization criteria. Additionally, ETR uses the entire original dataset as training data (to construct leaf nodes), whereas RF uses bootstrap sampling to create subsets of data. The simpler node branching method in ETR makes it computationally more efficient than other ensemble methods. The higher randomness in ETR reduces the overfitting problem, while the use of the entire dataset minimizes bias and improves the model’s performance for new data.

**Fig. 4:**
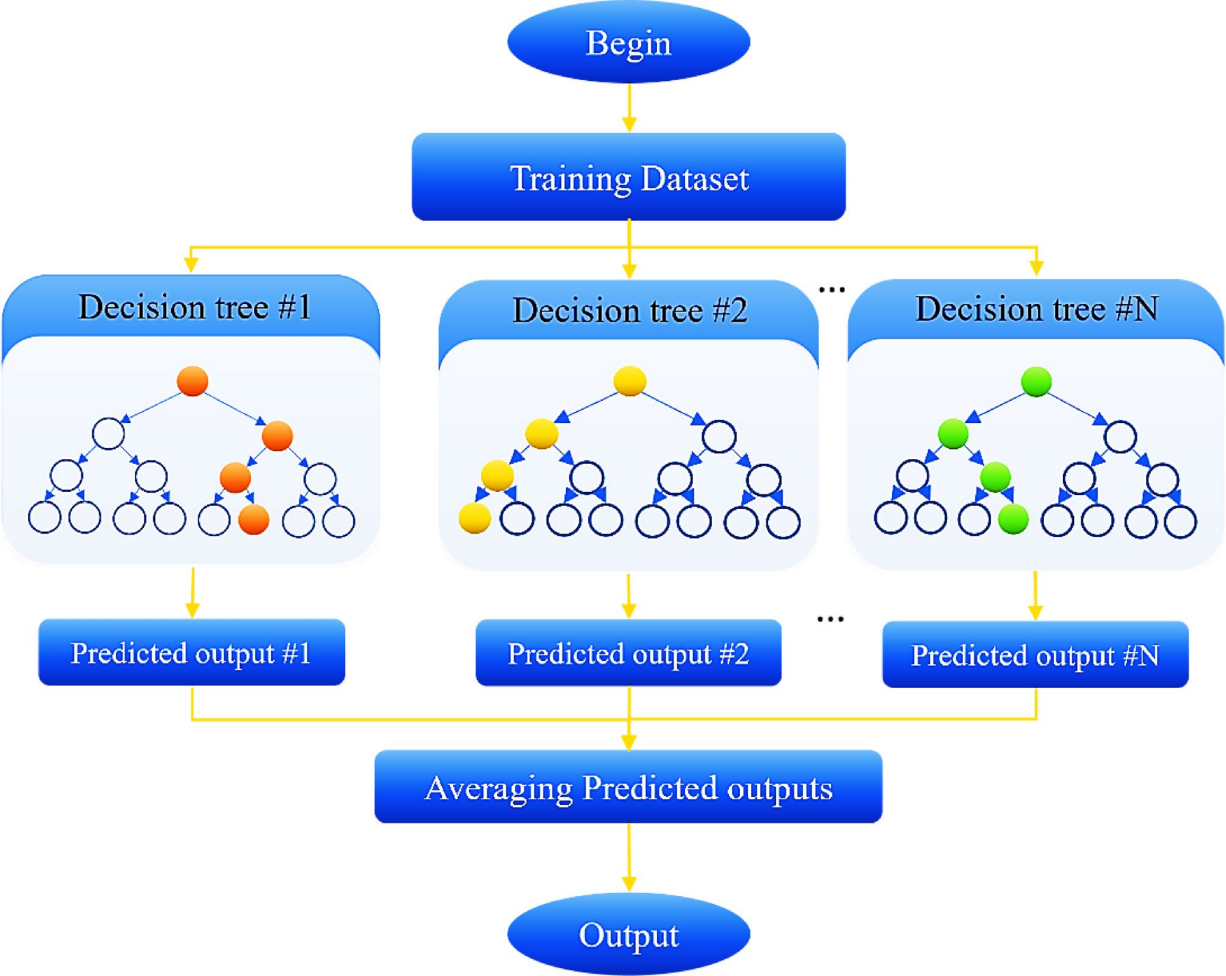
ExtraTrees flowchart.

To enhance performance, several hyperparameters are tuned for both RF and ETR. These hyperparameters include the number of trees, the maximum depth of each tree, the number of features considered at each branching, the minimum number of samples required for branching, and the minimum number of samples required to split leaf nodes^62^. Adjusting these hyperparameters allows for balancing bias and variance, thereby improving the model’s prediction performance (Fig. 4).

#### Machine learning methods modeling process

The modeling process using ML algorithms involves a series of structured steps, progressing from data preparation to model evaluation and optimization. The first step is data collection and preprocessing. In this stage, the data must be examined for quality and suitability for the problem at hand. Subsequently, actions such as removing outliers, filling in missing values, and standardizing the data to create a uniform scale are performed. The use of algorithms like CatBoost, which can directly process categorical data, reduces the complexity of this stage.

Next, key feature selection and engineering are carried out, as these features directly influence the model’s performance. In this step, tools such as correlation analysis and dimensionality reduction methods, like Principal Component Analysis (PCA), are used to identify and select the most impactful features. This process helps reduce data complexity and increases processing speed. Once the data is prepared, the appropriate algorithm for modeling is chosen. The selection of the algorithm depends on the type of data and the model’s objective. Algorithms like RF, ETR, and CatBoost, due to their various capabilities, are suitable options for diverse problems.

Fine-tuning hyperparameters, such as the number of trees and their depth, through methods like grid search or random search, ensures improved model accuracy and establishes a balance between bias and variance. After model tuning, training begins using the training data, and performance is evaluated using validation data. Techniques like cross-validation help mitigate overfitting and ensure the model’s performance on new data. Models like AdaBoost, which focus on difficult samples and correct errors at each stage, yield better prediction results.

In the final stage, models are evaluated and compared using metrics such as MSE, MAE, and the Coefficient of Determination (R^2^). Algorithms like ETR, which utilize randomization in the branching process and employ the entire dataset, and CatBoost, with its ability to directly handle categorical data, have shown successful performance in many complex problems. These steps aid in selecting the optimal model and significantly increase prediction accuracy.

## Results and discussion

As stated in the data collection section, this study was conducted to investigate the CO_2_ and CH_4_ adsorption capacities in tight reservoirs using available experimental data and ML techniques. The dataset comprises 3,804 samples of measured parameters, including temperature, pressure, rock type (shale and coal), TOC, moisture content, and the percentage of CO_2_ in the injected gas. These data were statistically evaluated, and the results were analyzed using graphical charts such as violin plots, pair plots, and heat maps, which illustrate the dispersion, continuity, and correlation among variables.

The dataset used in this study is divided into two sections: CO_2_ and CH_4_ adsorption data. Each section was analyzed separately and then compared. Both sections were further divided into two subsets: a training set containing 70% of the data and a testing set comprising the remaining 30%. The training dataset was utilized to develop the most optimal model and select relevant features. During this stage, the model learned to establish relationships between the input parameters and approximate the target values to the actual or measured values.

During training, the model adjusted its parameters (such as weights in neural networks) to minimize prediction errors, enabling it to make accurate predictions based on the training data. The testing dataset, which accounted for 30% of the total data, was used to evaluate the predictive capability and performance of the trained model. After training, the model’s performance was assessed using the test data without further adjustments to its parameters. This approach demonstrated that the model effectively avoided overfitting on the training data while achieving acceptable performance.

The model performed consistently well on both the training and testing datasets, producing satisfactory results and validating its performance. This indicates that the model is robust and likely to perform well in real-world scenarios.

### Hyperparameter optimization

This study addresses the challenge of parameter tuning in ML algorithms and proposes Bayesian optimization as an effective solution to this problem. ML algorithms often require the adjustment of parameters to control the learning rate and model capacity, which can be considered a nuisance. While one approach is to minimize the need for these parameters, another approach is to automate their optimization. Bayesian optimization is recommended as an efficient method for this purpose, as it has demonstrated superior performance compared to other global optimization techniques.

This method operates under the assumption that the unknown function (in this case, the performance of a learning algorithm with various parameter settings) is sampled from a Gaussian process, which maintains a posterior distribution. The optimization process involves selecting parameters for subsequent evaluations based on criteria such as the expected improvement (EI) or the upper confidence bound (UCB) derived from the Gaussian process. Studies have shown that EI and UCB are highly effective in identifying global optima for many black-box functions^63–66^.

The distinctive characteristics of ML algorithms in optimization are further elaborated in this study. To evaluate each function, the time variations caused by differences in model complexity are analyzed, along with the economic implications of performing experiments on cloud computing platforms. According to the study by Snoek and Larochelle^67^, Bayesian optimization algorithms have demonstrated favorable results in ML applications.

This research also advocates for a fully Bayesian treatment of the Gaussian process kernel, rather than merely optimizing its hyperparameters. Furthermore, the aforementioned study introduces new algorithms designed to account for variable experimental costs or the simultaneous execution of experiments. Gaussian processes are highlighted as effective alternative models in such scenarios.

In this study, the selection of hyperparameters played a critical role in enhancing the performance and accuracy of models used for analyzing laboratory data. Table 2 presents the optimal hyperparameter settings for four different models— RF, CatBoost, Extra Trees, and AdaBoost—applied to the adsorption of CO_2_ and CH_4_ gases. These settings were determined using the Bayesian optimization method.

**Table 2:** Optimal hyperparameter Settings.

CO_2_	Random forest	max depth	101
		min samples leaf	1
		min samples split	2
		n estimators	618
	CatBoost	depth	8
		l2 leaf reg	2.8601
		learning rate	0.155
	Extra trees	max depth	14
		min samples leaf	1
		min samples split	2
		n estimators	62
	AdaBoost	learning rate	0.9855
		max depth	9
		n estimators	140
CH_4_	Random forest	max depth	179
		min samples leaf	1
		min samples split	2
		n estimators	149
	CatBoost	depth	8
		l2 leaf reg	2.4742
		learning rate	0.1857
	Extra trees	max depth	20
		min samples leaf	1
		min samples split	2
		n estimators	258
	AdaBoost	learning rate	1
		max depth	10
		n estimators	57

For the CO_2_-related data, the optimal hyperparameters were determined as follows:

- **Random Forest**: The maximum tree depth is set to 101, and the minimum number of samples per leaf is 1, enabling the model to capture more complex patterns. The minimum number of samples for splitting nodes is 2, and the total number of trees is 618, enhancing both accuracy and robustness.
- **CatBoost**: The tree depth is 8, with an L2 regularizer value of 2.8601 for the leaves. A learning rate of 0.155 ensures a balance between convergence speed and model accuracy.
- **Extra Trees**: The model is configured with a maximum tree depth of 14 and 62 trees, optimizing computational efficiency while maintaining performance.
- **AdaBoost**: With a high learning rate of 0.9855, a tree depth of 9, and 140 learners, the model achieves faster convergence and reliable results.

For the methane-related data, the hyperparameter settings were adjusted to manage complexity and improve predictive performance:

- **Random Forest**: A maximum tree depth of 179 captures finer details in the data, while 149 trees contribute to enhanced accuracy.
- **CatBoost**: The tree depth is 8, with an L2 regularizer value of 2.4742. A learning rate of 0.1857 ensures an optimal trade-off between accuracy and convergence speed.
- **Extra Trees**: To uncover more intricate patterns, the model is designed with a maximum tree depth of 20 and 258 trees.
- **AdaBoost**: A learning rate of 1, combined with a tree depth of 10 and 57 learners, results in fast convergence and effective performance in analyzing methane data.

The careful selection of hyperparameters for these models— RF, CatBoost, Extra Trees, and AdaBoost—has significantly enhanced their accuracy and robustness in analyzing laboratory data for CO_2_ and methane. The tailored combination of settings, such as tree depth, learning rate, and the number of trees, has allowed each model to operate optimally based on the specific characteristics of the dataset. These configurations strike a precise balance between capturing complex patterns, achieving convergence speed, and minimizing overfitting, ultimately leading to improved data evaluation and more accurate analysis in related studies.

### Evaluation and model performance

#### Error metrics

Evaluation metrics play a vital role in assessing the performance of ML models. They provide a means to measure, analyze, and improve the models’ accuracy and operational capabilities. Selecting the appropriate evaluation metrics is crucial, as decisions based on these metrics can significantly influence the quality and performance of ML models. Hence, careful consideration of the evaluation metrics for each specific ML task or project is essential.

One of the most widely used evaluation metrics is the coefficient of determination (R^2^), which quantifies how much of the variance in the dependent variable is explained by the model. A higher R^2^ value indicates a stronger fit between the model and the data, with a value of 1 representing a perfect fit and 0 indicating no explanatory power (Eq. 1). This metric is particularly effective in illustrating the degree of difference between the actual and predicted values of the model.

Another key metric used in this study is the RMSE, which measures the square root of the mean squared difference between the actual and predicted values (Eq. 2). RMSE is highly sensitive to outliers and provides insights into the model’s overall prediction accuracy, with lower values indicating greater precision.

The MAE is also utilized to evaluate model performance. MAE calculates the average absolute difference between actual and predicted values, providing a straightforward interpretation of prediction errors in the same unit as the target variable (Eq. 3). Like RMSE, a lower MAE value reflects better model performance.

Additionally, the Mean Absolute Percentage Error (MAPE) is used as a performance metric to measure prediction accuracy in percentage terms. It is computed by dividing the absolute difference between predicted and actual values by the actual values and multiplying the result by 100. MAPE is particularly valued for its simplicity and interpretability, with lower values signifying higher accuracy. However, MAPE has limitations when applied to datasets containing values close to zero, as the percentage error can become exaggerated.

In conclusion, selecting and employing the right evaluation metrics, such as R^2^, RMSE, MAE, and MAPE, is integral to understanding and improving ML models’ performance. Each metric provides unique insights into the model’s accuracy and operational effectiveness, making them essential tools in developing reliable ML solutions.

1 $$\:{R}^{2}=1-\frac{\sum\:_{i=1}^{N}{\left(({CO2,CH4\:sorption)}_{i}^{exp}-{(CO2,CH4\:sorption)}_{i}^{pred}\right)}^{2}}{\sum\:_{i=1}^{N}{\left({(CO2,CH4\:sorption)}_{i}^{exp}-\overline{{{(CO2,CH4\:sorption)}^{exp}}}\right)}^{2}}$$

2 $$\:RMSE=\:\sqrt{\frac{\sum\:_{i=1}^{N}{{\left(\right(CO2,CH4\:sorption)}_{i}^{exp}-{(CO2,CH4\:sorption)}_{i}^{pred})}^{2}}{N}}$$

3 $$\:MAE=\:\frac{1}{N}\sum\:_{i=1}^{N}\left|{\left(\right(CO2,CH4\:sorption)}_{i}^{exp}-{(CO2,CH4\:sorption)}_{i}^{pred})\right|$$

4 $$\:MAPE=\:\frac{100}{N}\sum\:_{i=1}^{N}\left|\frac{{\left(\right(CO2,CH4\:sorption)}_{i}^{exp}-{\left(CO2,CH4\:sorption\right)}_{i}^{pred})}{{\left(CO2,CH4\:sorption\right)}_{i}^{exp}}\right|$$

These equations (1 to 4) contain values presented in Table 3. The values obtained from the calculations and the associated errors in the data are provided in Table 4. Subsequently, the predictive capability of the model, along with a discussion of the results presented graphically, is evaluated.

**Table 3:** Introduction of parameters for error evaluation equations.

Predicted Values by the Model	$\:{(CO2,CH4\:sorption)}_{i}^{pred}$
Experimental Values	$\:({CO2,CH4\:sorption)}_{i}^{exp}$
Mean Values	$\overline{{{(CO2,CH4\:sorption)}^{exp}}}$
Number of Data Points	N
Iteration	i

For model evaluation, various error metrics, including the coefficient of determination, RMSE, MAE, and MAPE, were calculated for both the test data and the entire dataset. The results are shown in Table 4.

**Table 4:** Calculations and error Report.

Error metric	Dataset	CO_2_				CH_4_			
		Random forst	CatBoost	Extra trees	AdaBoost	Random forest	CatBoost	Extera trees	AdaBoost
R ^2^	Train	0.9968	0.9999	1.0000	0.9985	0.9971	0.9985	0.9998	0.9970
	Test	0.9903	0.9968	0.9946	0.9877	0.9796	0.9911	0.9873	0.9742
	Total	0.9947	0.9989	0.9982	0.9950	0.9923	0.9965	0.9963	0.9907
RMSE	Train	0.2709	0.0519	0.0243	0.1843	0.0688	0.0487	0.0183	0.0702
	Test	0.5052	0.2921	0.3756	0.5681	0.1722	0.1140	0.1359	0.1936
	Total	0.3579	0.1660	0.2070	0.3476	0.1185	0.0746	0.0760	0.1213
MAE	Train	0.1611	0.0364	0.0095	0.1224	0.0378	0.0346	0.0074	0.0487
	Test	0.3381	0.2148	0.2716	0.3934	0.0980	0.0726	0.0781	0.1145
	Total	0.2144	0.0901	0.0883	0.2039	0.0558	0.0460	0.0286	0.0685
MAPE	Train	8.8524	3.4402	0.8646	28.0617	6.3432	5.8858	0.9053	12.2420
	Test	22.5793	17.8237	19.6057	32.9776	20.6930	25.2515	22.2115	31.1199
	Total	12.9831	7.7684	6.5042	29.5410	10.6515	11.7000	7.3021	17.9097

By evaluating the error metrics, it can be observed that the performance of all four developed models for both CO_2_ and CH_4_ adsorption is very good and similar to each other. Since the performance on the test data did not show a significant decline compared to the training data in all the constructed models, it can be concluded that overfitting did not occur. The CatBoost and Extra Trees models performed better than the other models across all error metrics. The correlation coefficient and RMSE for the CatBoost model, for both gases and across all data, are better than those of Extra Trees. However, the MAE and MAPE values for the total data are more favorable in the Extra Trees model compared to CatBoost. It should be noted that although Extra Trees exhibited better performance than CatBoost in the training dataset for both gases, it also showed a more significant decline in the test dataset. Therefore, although Extra Trees performed better in terms of MAE and MAPE error metrics, it has lower generalizability compared to the CatBoost model overall.

#### Graphical methods

**Fig. 5:**
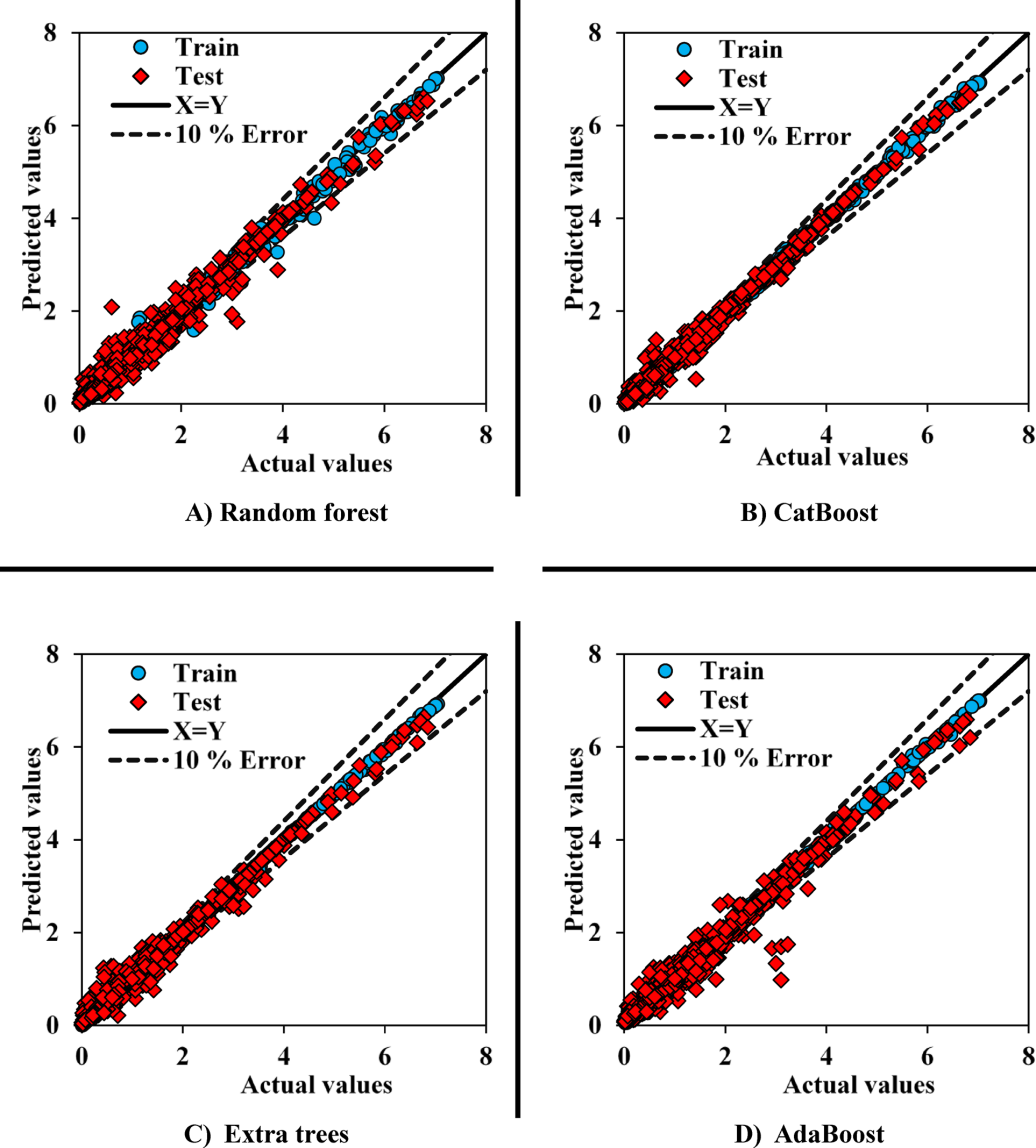
Shear plot - performance of implemented models in CH_4_ adsorption.

To gain a better understanding of the models’ performance, cross plots can be utilized. In this method, the model outputs are plotted against the actual values. The closer the points are to the line with a slope of one and an intercept of zero, the better the model’s performance. The performance of the models related to CH_4_ adsorption is shown in Fig. 5, where the superior performance of the CatBoost and Extra Trees models is evident. As expected from the error metrics, AdaBoost exhibited the worst performance, with many points showing significant deviations from the actual values. The RF model demonstrated relatively good accuracy, but there was still noticeable data dispersion compared to the ideal line. Although the Extra Trees model performed better than CatBoost in the MAE and MAPE error metrics, it is evident from the cross plot that the CatBoost model demonstrated much better performance, with the data points well aligned along the ideal line.

**Fig. 6:**
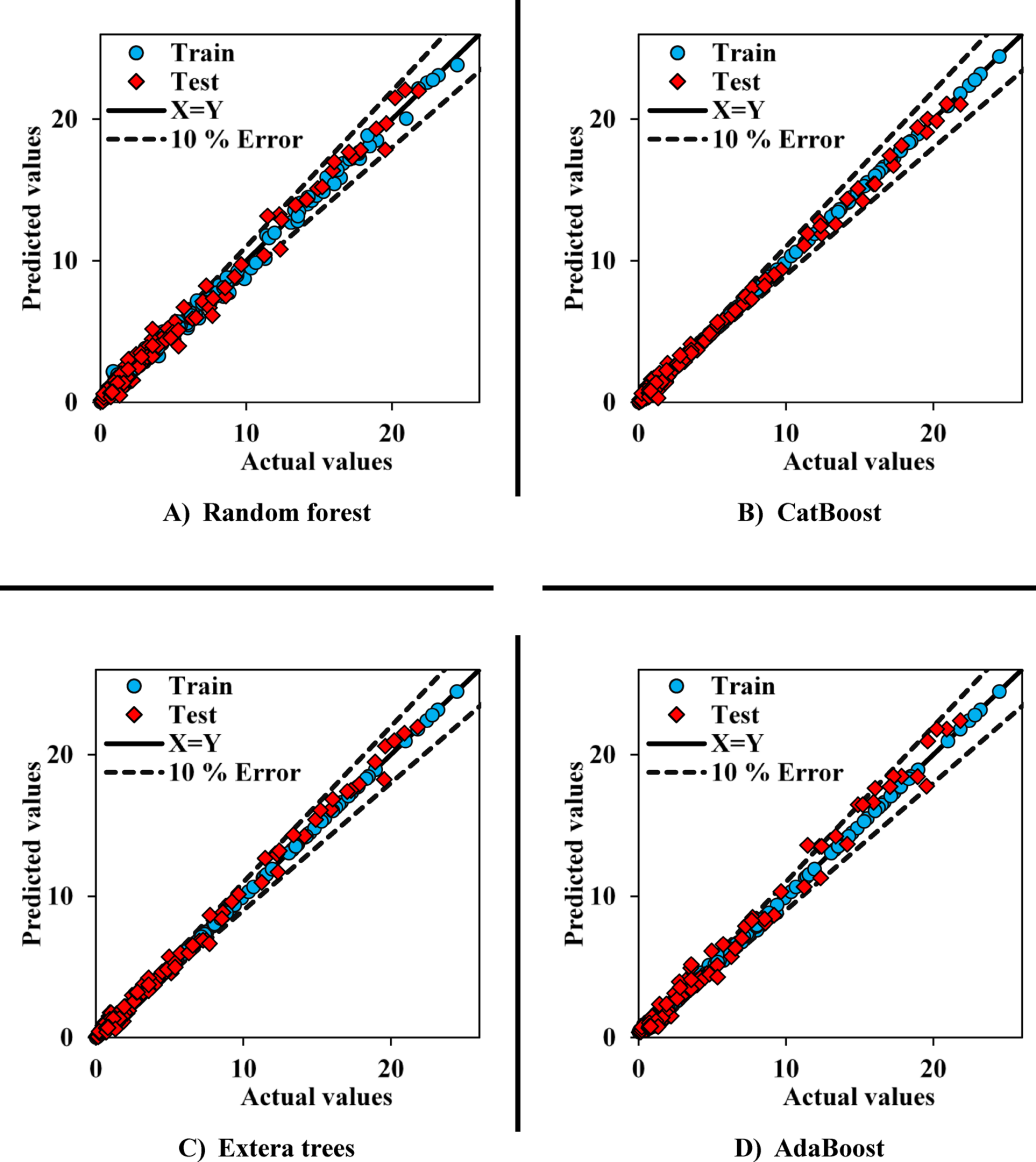
Cross plot - performance of implemented models in CO_2_ adsorption.

The cross plot for the CO_2_ models is also shown in Fig. 6. Here, the performance of the two models, AdaBoost and RF, is weaker compared to the other models. As with the CH_4_ models, despite Extra Trees performing better than CatBoost in the MAE and MAPE error metrics, the cross plots show the superior performance of CatBoost compared to Extra Trees.

**Fig. 7:**
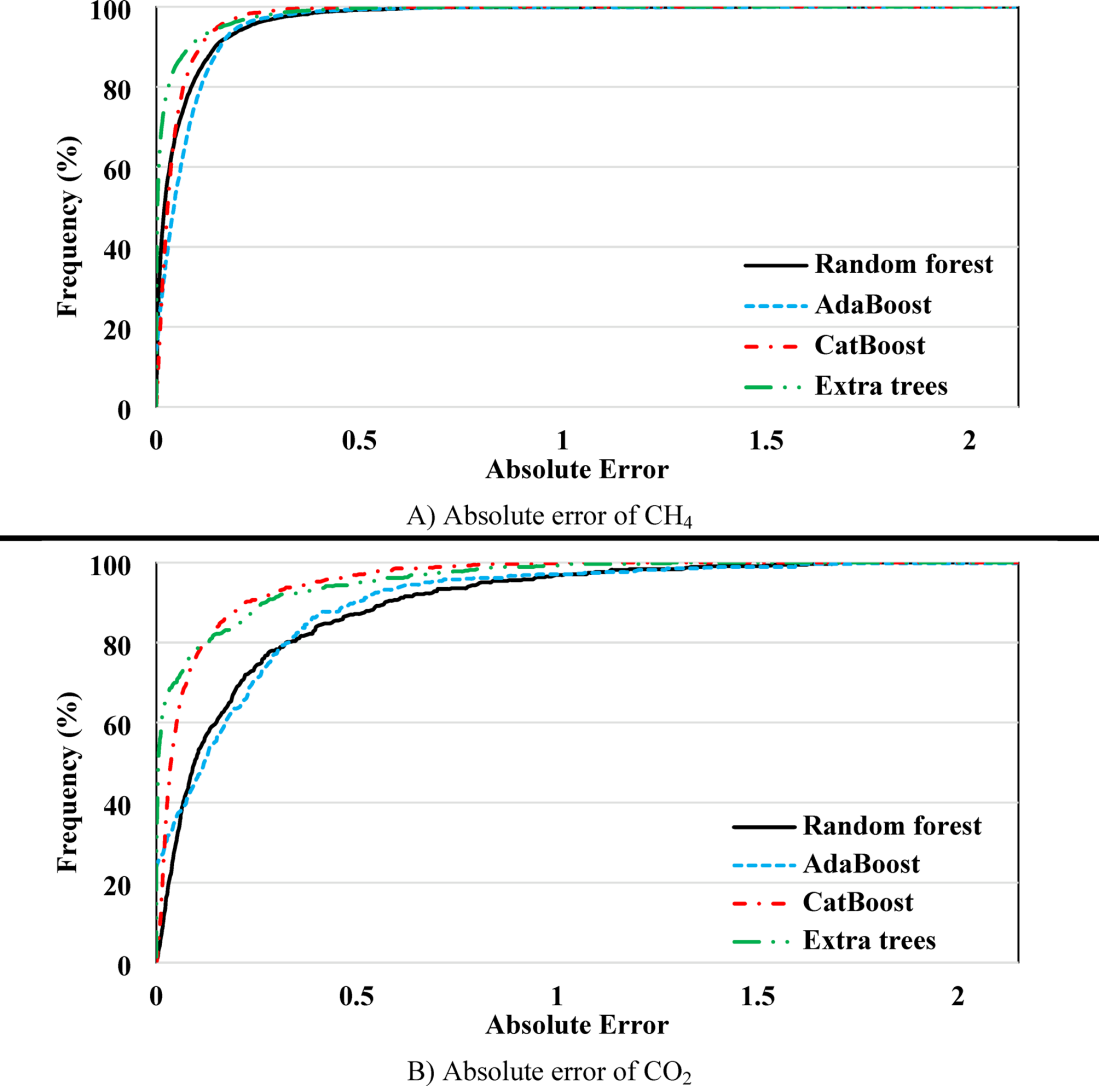
Model performance evaluation based on the Cumulative Frequency chart for assessing absolute error values.

To closely examine the model performance, the cumulative frequency chart for the absolute error of each model is shown in Fig. 7. In this approach, the higher the chart for a model, the better its performance. Figure 7 A corresponds to the models built for CH_4_. Based on this, the Extra Trees model outperforms the others noticeably up to an absolute error of 0.14, but after that, the CatBoost model performs better. These two models estimated 94.2% of the data with an error of less than 0.14, indicating their exceptional performance. The RF and AdaBoost models show similar performance, with RF outperforming up to an error of 0.17. However, after that, AdaBoost improves and shows better performance.

To enhance the transparency and reproducibility of this study, the training/testing datasets and output results associated with the CatBoost model—used for evaluating CO_2_ and CH_4_ adsorption—have been provided as supplementary materials accompanying this manuscript. 

The cumulative frequency chart for CO_2_ also shows similar results to CH_4_ (Fig. 7B). However, in this case, the superior performance of the CatBoost and Extra Trees models compared to RF and AdaBoost is clearly noticeable, with a significant gap between the charts. Both Extra Trees and CatBoost models estimated 80% of the data with an absolute error of less than 0.13. Additionally, the CatBoost model estimated over 90% of the data with an absolute error of less than 0.22, while this value for the Extra Trees model reaches 0.27. The results of this section indicate that, despite the better performance of Extra Trees compared to CatBoost in terms of MAE and MAPE, both models are highly competitive, with CatBoost showing superior performance in some cases.

### Outlier detection

To identify outliers and the applicable range of the model within a dataset, a well-known graphical method called the Williams plot was used. Utilizing the Williams plot and identifying outliers in this chart can assist in evaluating the reliability of the resulting model. In fact, a high percentage of outliers can disrupt the model’s performance and ultimately render it unreliable. In other words, the significant presence of outliers can lead the model to focus unduly on data points that are statistically invalid, thus compromising its overall performance. Therefore, the examination and identification of outliers is a critical step in modeling.

This technique relies on the Hat matrix (H) and the calculation of standardized residuals (SR). The Hat matrix is used to calculate the predicted values of the response variable, while the standardized residual is the residual divided by its estimated standard error. The matrix MX has dimensions of n × p, where n and p represent the number of data points and input variables, respectively, and SD is the standard deviation.

Leverage points, which are data points that have a significant effect on the regression coefficients of the model, can be identified using the Hat matrix. The diagonal elements of the Hat matrix are examined to identify these leverage points. Data points with a value greater than the leverage warning value (Hat*) = 3(*p* + 1)/n are considered high-leverage points.

The safe ranges for statistical validation of both the developed models and the dataset are 0 ≤ H ≤ H* and − 3 ≤ SR ≤ 3. If data points do not match the defined ranges, they can be categorized into three possible groups:

1. Suspicious vertical data: This includes data points that fall outside the ranges Hat* ≥ H and SR > 3 or SR < −3, and are outside the applicable range.
2. Good leverage data: This includes data points that fall within the ranges Hat* < H and − 3 ≤ SR ≤ 3.
3. Bad leverage data: This includes data points that fall within the ranges H > Hat* and SR > 3 or SR < −3.

**Fig. 8:**
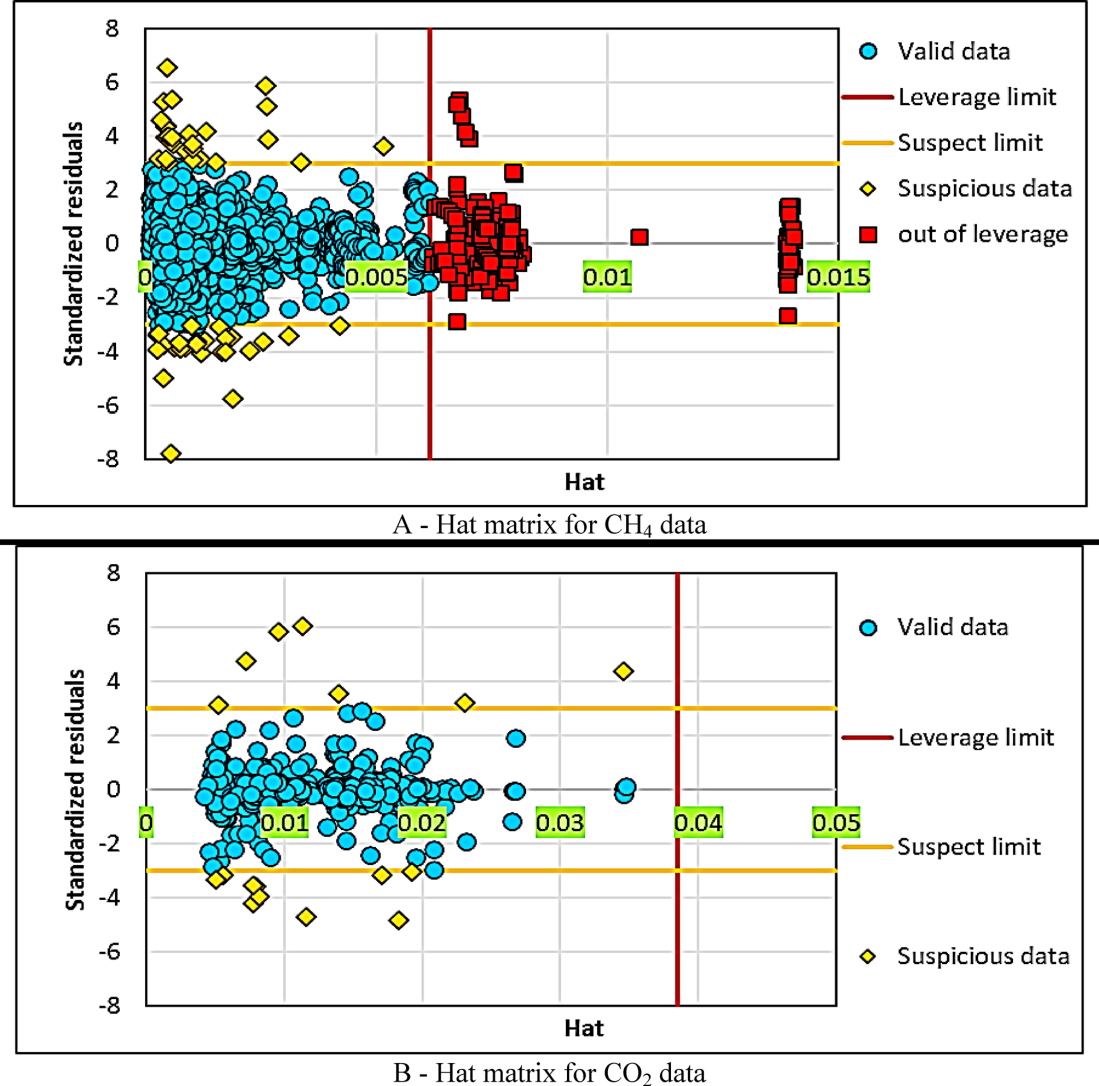
Identification of outliers and model applicability range using the Williams plot.

As shown in Fig. 8, the first chart (A) examines the data related to CH_4_ adsorption using the Hat matrix. The horizontal axis represents the Hat values, while the vertical axis indicates the SR. Blue points are identified as valid data and fall within the red line (leverage threshold). Yellow points, which are near the suspicious range, are identified as suspicious data. In this chart, a significant number of data points lie within the suspicious range or outside of leverage (red points), indicating the potential presence of outliers or points with a significant impact on the model.

Leverage thresholds of 0.005, 0.01, and 0.015 are set to help identify high-risk data points. This analysis highlights the importance of monitoring the data to ensure modeling accuracy. In the second chart (B), valid data are marked with blue points, while suspicious data are marked with yellow points. The proportion of suspicious data is lower compared to the CH_4_ chart, indicating more stable data for CO_2_ in modeling.

The Hat values are divided into ranges of 0.01, 0.02, 0.03, 0.04, and 0.05, which are used for a more detailed analysis of the impact of different data points on the modeling. Overall, this chart shows that CO_2_ data has less impact outside the leverage threshold, and the model potentially performs better in this region. These analyses emphasize the impact of outliers in the modeling of CH_4_ and CO_2_ adsorption in reservoirs and highlight the need for careful data examination to improve model accuracy.

Using a leverage threshold of 0.0062 and |SR| > 3, the CH₄ dataset contained outliers that exhibited significant deviations in multiple input features. A statistical comparison revealed that:

Results in Table 5 indicate that the outliers are characterized by extremely high TOC and elevated moisture content, suggesting that they may reflect valid but rare geological formations (e.g., highly organic-rich, water-retentive shales). These points could also stress the limitations of the model in high-TOC, high-moisture regions.

**Table 5:** Average value of each parameter in whole dataset and outliers for CH_4_.

Feature	Mean (All Data)	Mean (Outliers)
TOC (%)	10.22	40.00
Moisture (%)	0.92	2.22
Temperature (°C)	57.45	51.17
Pressure (MPa)	11.45	8.31

For the CO_2_ dataset, with a leverage threshold of 0.0385 and |SR| > 3, the outliers were found to differ primarily in terms of TOC only. The statistical summary is shown below:

Based on Table 6, Unlike CH_4_, CO_2_ outliers are not high in moisture; in fact, they have significantly lower moisture content than the average. This suggests that the model underperforms in low-moisture and high-TOC conditions, which are geologically plausible scenarios such as dry, highly mature shales. These outliers do not show high leverage and are unlikely to distort the model structurally, indicating they are likely true but difficult-to-predict samples rather than data errors.

**Table 6:** Average value of each parameter in whole dataset and outliers for CO_2_.

Feature	Mean (All Data)	Mean (Outliers)
TOC (%)	50.14	70.74
Moisture (%)	1.02	0.24
Temperature (°C)	48.18	45.24
Pressure (MPa)	8.70	11.19

### Sensitivity analysis

**Fig. 9:**
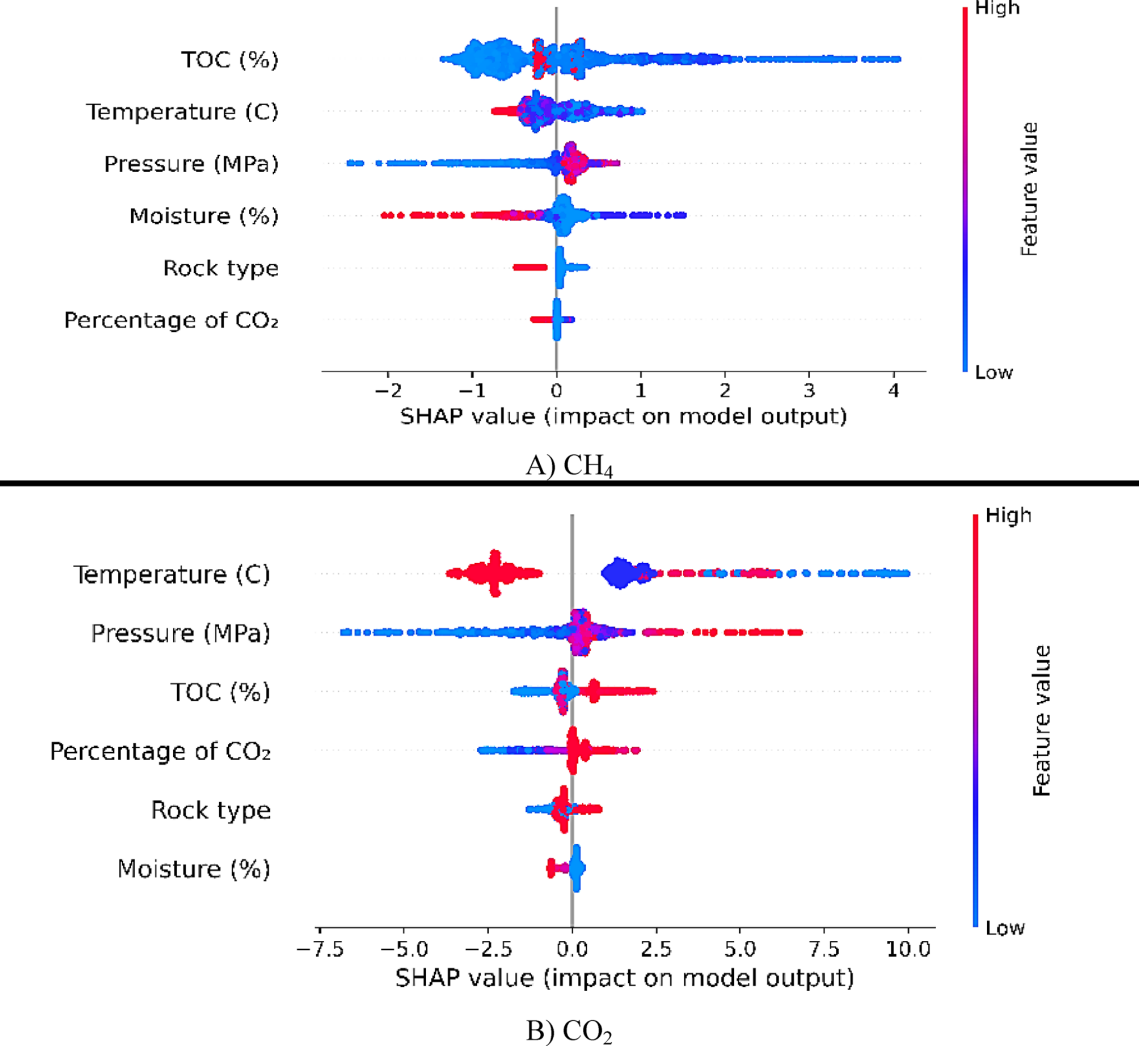
Sensitivity analysis based on the SHAP technique.

Sensitivity analysis is one of the key steps in improving model performance and interpreting the results in the prediction of CO_2_ and CH_4_ gas adsorption. This method helps identify the impact of input variables on the model’s outcomes and indicates which parameters have the greatest influence on model performance. In this study, sensitivity analysis was performed using ML algorithms such as CatBoost and Extra Trees, based on the SHAP (Shapley Additive Explanations) technique (Fig. 9).

The use of the SHAP technique allows for the examination of the contribution of each input variable to the model’s output. This technique not only reveals the impact of variables at different levels but also uncovers the nonlinear relationships and interactions between parameters. The SHAP analysis demonstrated that the pressure variable had the greatest contribution to the prediction of gas adsorption capacity at all stages of modeling, while the temperature variable only had a significant impact at high values.

According to the SHAP chart for CH_4_ gas, the pressure variable and the percentage of TOC are the most influential factors on CH_4_ adsorption. An increase in pressure significantly enhances CH_4_ adsorption capacity, as indicated by a high positive SHAP value. The percentage of TOC also shows a similar positive influence, reflecting the importance of the organic content of the reservoir rock in improving CH_4_ storage. In contrast, temperature has a negative impact on CH_4_ adsorption. Higher temperatures result in a reduced storage capacity, which is observed in the lower SHAP values at higher temperatures. This can be attributed to the decreased tendency of CH_4_ molecules to adsorb on the rock surface at higher temperatures. Other variables, such as rock type and moisture, also have limited but significant impacts on CH_4_ adsorption. Rock type, due to its porosity and structural characteristics, may enhance adsorption, while increased moisture negatively affects CH_4_ adsorption, resulting in a reduced SHAP value.

For CO_2_ gas, pressure remains the most influential variable. Increased pressure leads to a significant rise in the SHAP value, indicating an increased CO_2_ adsorption capacity at higher pressures. Additionally, the percentage of CO_2_ in the gas mixture has a positive and significant impact, with higher values of this variable improving CO_2_ adsorption. Temperature, like for CH_4_, has a negative effect on CO_2_ adsorption. The negative impact of temperature on CO_2_ is more pronounced than for CH_4_, and the decrease in adsorption capacity with rising temperature is clearly visible in the SHAP chart. This result may be due to the higher volatility of CO_2_ at elevated temperatures. Variables such as rock type and moisture play secondary roles in CO_2_ adsorption. Although their impact is limited, rock type, due to its surface characteristics and porosity, and moisture, due to occupying pore space, can influence the final results. This sensitivity analysis for CO_2_ provides valuable insights for the design and optimization of storage systems.

In general, sensitivity analysis is an effective tool for gaining a deeper understanding of the impact of various variables on gas adsorption. This method not only aids in improving modeling accuracy but also provides useful information for designing future experiments and optimizing operational conditions in gas adsorption systems.Unlike recent studies such as Tavakolian et al.^1^, which evaluated a broad range of machine learning techniques for modeling CH_4_ and CO_2_ sorption capacity and identified Random Forest as the most accurate predictor, the present study introduces a unified and systematic benchmarking framework for multiple ensemble-based algorithms, including Random Forest, CatBoost, AdaBoost, and Extra Trees. All models are implemented using identical data partitioning, preprocessing, and evaluation protocols, with Bayesian hyperparameter optimization consistently applied across the entire modeling pipeline. This standardized framework minimizes biases associated with unequal tuning strategies and enables a fair comparison of intrinsic model capabilities.

Beyond predictive accuracy, this study places stronger emphasis on interpretability and physical consistency by employing SHAP-based sensitivity analysis to quantify the influence of key parameters on CO_2_ and CH_4_ adsorption. While the feature importance trends reported by Tavakolian et al.1 are generally confirmed—particularly the dominant roles of pressure and TOC—the present analysis provides deeper insights into competitive adsorption mechanisms, revealing that pressure and CO_2_ concentration enhance CO2 adsorption while exerting an inverse effect on CH_4_ due to site competition. Moreover, although Extra Trees achieves lower MAE and MAPE values in certain cases, the CatBoost model demonstrates superior generalization performance and stability on unseen data, achieving R^2^ values of 0.9989 for CO_2_ and 0.9965 for CH_4_. These findings highlight important trade-offs between accuracy and robustness and offer practical guidance for selecting reliable machine learning tools for gas adsorption modeling in heterogeneous tight reservoirs.

## Conclusions

This study demonstrates the significant potential of ML models in enhancing the understanding of CO_2_ and CH_4_ adsorption capacities in tight reservoirs, utilizing data from prior research. By integrating ML techniques with laboratory data, the research provides valuable insights into optimizing gas injection and storage processes. The evaluation of 3,804 data points, covering variables such as temperature, pressure, rock type, TOC, and gas composition, revealed that different parameters exert varying effects on adsorption capacity.

This study underscores the value of data-driven modeling for adsorption analysis in tight reservoirs by utilizing a diverse and extensive dataset in conjunction with state-of-the-art machine learning techniques. Among the evaluated algorithms, ensemble-based models such as Random Forest, CatBoost, AdaBoost, and Extra Trees demonstrated superior predictive capability and interpretability. These results highlight the importance of employing advanced ML tools to uncover complex patterns in experimental data, ultimately improving our understanding of gas-rock interactions under varying thermodynamic conditions. The findings contribute to the development of more accurate predictive tools for gas storage and enhanced recovery strategies in unconventional reservoirs.

The study found that CO_2_ percentage in injected gas and TOC are pivotal factors influencing CO_2_ adsorption, with TOC positively impacting CO_2_ adsorption by providing microporous sites. Pressure also plays a critical role, enhancing CO_2_ adsorption while inversely affecting CH_4_ adsorption due to competitive interactions. Temperature had a negative impact on CO_2_ adsorption but slightly increased CH_4_ adsorption, suggesting gas-specific interactions with rock properties. Correlation analysis further confirmed the competition between CO_2_ and CH_4_ for adsorption sites, with TOC and CO_2_ concentration demonstrating the strongest positive effects on CO_2_ adsorption.

ML models, particularly CatBoost and Extra Trees, proved highly effective in predicting gas adsorption, achieving high R^2^ values (0.9989 for CO_2_ and 0.9965 for CH_4_) and low prediction errors (RMSE and MAE). The CatBoost model demonstrated superior overall performance, with strong stability and accuracy across all metrics. The sensitivity analysis revealed that pressure is the most influential factor, followed by TOC and CO_2_ percentage, while temperature acted as a restrictive variable. Secondary variables such as rock type and moisture content, though less impactful, were also highlighted.

The results underline the importance of careful hyperparameter tuning and the application of advanced ML techniques to improve model performance and optimize gas storage systems. This research provides a robust framework for future studies on gas adsorption in diverse reservoir conditions, emphasizing the utility of combining laboratory data with ML methods. The findings offer practical guidance for managing gas injection processes and improving storage capacity in tight reservoirs.

### Challenges ahead

- Generalizability of Results

Although the CatBoost model has demonstrated significant performance, the generalization of these models to other reservoir conditions and unseen data still requires further investigation. Particularly, the behavior of gases may differ across various reservoirs or under different operational conditions.

- Use of Advanced and Interpretable Models

The use of more advanced methods and models can significantly contribute to modern research fields. While ML techniques were utilized in this study, other methods such as deep learning or interpretable models like Genetic Programming (GP), GEP, and Group Method of Data Handling (GMDH) could be considered for future research.

- Recommendations

Future studies could explore several capabilities and potentials to expand the scope of this research, making it more comprehensive and detailed, and thus more accessible to both the scientific and industrial communities. Among these considerations are the use of more extensive datasets, the application of novel ML techniques, and the integration of deep learning models. Additionally, the use of other gas mixtures, such as cushion gas, could be explored, particularly in reservoirs with different rock types and thermodynamic conditions.

## Data Availability

The datasets used and/or analyzed during the current study available from the corresponding author on reasonable request.

## Abbreviations

ANN: Artificial neural networks
CART: Classification and regression trees
CBM: Coalbed methane
CO_2_: Carbon dioxide
DT: Decision tree
DTR: Decision tree regression
EI: Expected improvement
ESGR: Enhanced shale gas recovery
ETR: Extra trees regressor
GBDT: Gradient-boosted decision tree
GEP: Genetic expression programming
GMDH: Group method of data handling
GP: Genetic programming
GPR: Gaussian process regression
GRNN: General regression neural network
GWO: Grey wolf optimization
H: Hat
LSSVM: Least squares support vector machines
MAE: Mean absolute error
MAPE: Mean absolute percentage error
ML: Machine learning
MSE: Mean squared error
PCA: Principal component analysis
RBFNN: Radial basis function neural network
RF: Random forest
RMSE: Root mean square error
SR: Standardized residuals
SVR: Support vector regression
TOC: Total organic carbon
UCB: Upper confidence bound
XGBoost: Extreme gradient boosting
