# Author Correction: Machine learning analysis of CO_2_ and methane adsorption in tight reservoir rocks

**DOI:** 10.1038/s41598-026-39397-4

**Published:** 2026-03-02

**Authors:** Mehdi Maleki, Mohammad Rasool Dehghani, Moein Kafi, Ali Akbari, Yousef Kazemzadeh, Ali Ranjbar

**Affiliations:** Department of Petroleum Engineering, Faculty of Petroleum, Gas, and Petrochemical Engineering, Persian Gulf University, Bushehr, Iran

Correction to: *Scientific Reports* 10.1038/s41598-025-10010-4, published online 08 July 2025

The Introduction, Data collection and specific description sections in the original version of the Article were incomplete.

The use of data originally characterized by Tavakolian et al. (Reference 1) was not fully justified or acknowledged. For the avoidance of doubt about the original contribution of the Article, explanation of data sourcing and methodology have been updated, and additional context about the study’s rationale has now been included.

Consequently, in the Introduction section,

“This study investigates the adsorption capacity of methane (CH_4_) and CO_2_ in tight reservoirs, specifically shale and coal, utilizing ML techniques. The research focuses on the application of ML algorithms to predict, evaluate, and optimize adsorption data for CH_4_ and CO_2_. The dataset was compiled from previous studies conducted by researchers in the field of underground hydrocarbon storage. Given the complexities involved in predicting gas behavior in unconventional reservoirs, this study holds significant importance. Traditional prediction methods, such as mathematical models, numerical simulations, and laboratory measurements, are often constrained by oversimplifications, high costs, and time-intensive processes. Consequently, ML techniques emerge as a promising alternative, offering higher accuracy and reducing computational complexity.”

now reads.

“This research focuses on modeling gas adsorption using machine learning in unconventional hydrocarbon reservoirs such as coal and shale using an experimental dataset published by Tavakolian et al.^1^. The authors provide a comprehensive dataset containing methane and carbon dioxide adsorption data from a variety of thermodynamic and geologic environments throughout the world. The authors have developed many machine learning models to estimate gas adsorption capacity based on pressure, temperature, rock type, TOC, moisture content and gas composition. This research intends on analyzing the unexplored machine learning techniques with one unified modeling framework. In this way, we can also compare the prediction quality versus the best-known machine learning prediction models outlined in previous works. Additionally, by performing a structured analysis of a variety of new machine learning approaches, we will provide an unbiased representation of the capabilities of current methods being used for predicting gas adsorption using current experimental data.”

Additionally, in the Data collection and specific description section,

“In this study, a dataset comprising 3,804 data points was utilized, originating from the comprehensive experimental compilation presented by Tavakolian et al.^1^. Specifically, the dataset includes 3,259 data points related to methane adsorption, 390 data points concerning CO₂ adsorption, and 155 data points for the co-adsorption of both gases. These data cover a broad range of thermodynamic conditions and incorporate essential variables such as temperature, pressure, rock type (shale and coal), total organic carbon (TOC), moisture content, and the percentage of CO₂ in the injected gas. This dataset enables a detailed evaluation of the influence of various parameters on gas adsorption capacity and facilitates a thorough understanding of gas behavior in different tight reservoir settings. Further details regarding the dataset and its development can be found in the literature review subsection of the Introduction.

A key aspect of this study was the selection of appropriate input variables for the ML models. These variables were chosen based on scientific analysis and reservoir engineering requirements to effectively reflect the influence of geological and operational factors on gas adsorption capacity. For instance, the percentage of CO_2_ in the injected gas was identified as one of the most critical variables, given its significant impact on the adsorption process. Additionally, other variables such as TOC and moisture content were incorporated into the modeling process, as each plays a crucial role in determining adsorption capacity.

To prepare the dataset for this study, raw data were collected from various sources, organized, and analyzed using Microsoft Excel. These data included variables such as pressure, temperature, rock type, and the composition of injected gases. The processed data were subsequently utilized as inputs for ML modeling techniques. To optimize the models, methods such as linear regression were employed, and the validity of the data was assessed and confirmed using the coefficient of determination (R^2^). The results of these analyses demonstrated a strong correlation between the input and output variables of the models. ML models for predicting gas adsorption capacity in reservoirs were developed based on the following relationships:

$${Capacity}_{Adsorption(CO2)}=f\left(Pressure,Temperature,TOC,Moisture,Percentage of C{O}^{2},Rock type\right)$$

$${Capacity}_{Adsorption(CH4)}=f\left(Pressure,Temperature,TOC,Moisture,Percentage of C{O}^{2},Rock type\right)$$

Equations (1) and (2) enabled researchers to accurately predict the effects of various parameters on gas adsorption capacity. Additionally, the models demonstrated the capability to forecast anomalous gas behaviors under high-pressure conditions. The findings of this study revealed that the proposed ML models, utilizing optimized input variables, are capable of accurately predicting gas adsorption capacities. Sensitivity analysis of the models further confirmed that parameters such as TOC and the CO_2_ fraction in the injected gas have the most significant impact on adsorption capacity. This research, by introducing innovative approaches for data analysis, provides a solid foundation for applying ML models in gas storage processes within unconventional reservoirs. Further details and statistical information related to this study are presented in Table 2.

The provided table contains various statistical details of data related to the excess adsorption of CO_2_ and CH_4_ gases, rock properties (such as TOC and moisture content), pressure, and temperature. Statistically, most of the data for parameters such as CO_2_ percentage, rock type, moisture content, and excess CO_2_ adsorption are concentrated at lower values, with their mode and median being zero, and their distribution showing a significant skew toward lower values (positive skewness). In contrast, parameters like TOC and excess CH_4_ adsorption exhibit distributions with moderate to high positive skewness, indicating a concentration of data at lower values. However, their maximum values are significantly higher than the mean and median, suggesting the presence of outliers or extreme values in the dataset.

On the other hand, parameters such as temperature and pressure have more balanced distributions, with their skewness generally being positive but low. Specifically, temperature, with a median of 50.4 °C and a mean of 57 °C, indicates a relatively uniform distribution across the temperature range. Overall, most of the data for rock properties and gases are concentrated at lower ranges, while higher values appear more scattered with distributions exhibiting high kurtosis (sharpness and peakedness), likely due to the presence of unusual data points or outliers. These results emphasize the importance of paying attention to outliers and extreme values in future analyses, particularly in the development of predictive and ML models.

After data collection, the data were examined and, from a statistical perspective, the CO_2_ and CH_4_ adsorption capacity was plotted as a function of temperature, pressure, rock type (shale and coal), TOC, moisture content, and the percentage of CO_2_ in the injected gas. In these analyses, violin plots, pair plots, and heat maps were presented.

In this study, a dataset comprising various features was collected and analyzed to investigate the characteristics of CO_2_ storage and gas behavior in different environments. Initially, violin plots (Fig. 1) were used to fully display the data distribution across various dimensions. These plots are particularly effective in showing the composition and scatter of the data, which is especially useful for analyzing complex and nonlinear data. Moreover, these plots specifically illustrate how the data are distributed across different levels for each feature. For instance, in the CO_2_ percentage plot, the data distribution is predominantly in the lower ranges, indicating the absence of high CO_2_ values in most samples; however, the spread of data towards higher values indicates variation among the samples. Similarly, the TOC distribution is mainly concentrated below 5%, which could be attributed to natural variations in rock composition and storage environments. Additionally, the moisture distribution has a broader range and greater scatter, reflecting significant differences in the moisture content of the samples. High variability is also observed in the temperature and pressure plots. Specifically, temperature spans from approximately 20 °C to 160 °C, allowing for the prediction of its effect on gas behavior and hydrogen storage characteristics. Pressure is primarily concentrated above 10 megapascals, indicating typical high-pressure gas storage conditions. Furthermore, CH_4_ and CO_2_ adsorption values are generally low, which may indicate storage environments with low adsorption of these gases. Overall, these plots provide a comprehensive picture of gas storage conditions and rock properties, serving as valuable tools for modeling analyses and engineering predictions in gas storage applications.

The paired plots in Fig. 2 illustrate the complex relationships between various parameters and the CO_2_ and CH_4_ adsorption capacities. Each individual plot analyzes the interaction between two specific variables and provides insights into their correlations and general trends in the data. One notable observation is seen in the plots showing the relationship between CO_2_ adsorption and pressure. As pressure increases, CO_2_ adsorption steadily increases, highlighting the significant impact of pressure on gas adsorption capacity in shale samples. This positive correlation suggests that higher pressures enhance the shale’s ability to adsorb CO_2_ through its pore network or adsorption mechanisms.

In contrast, the plots depicting the relationship between CH_4_ adsorption and pressure exhibit an inverse pattern. As pressure increases, CH_4_ adsorption decreases significantly, indicating an inverse relationship between pressure and methane adsorption. This observation suggests that higher pressures may disrupt the shale’s ability to retain CH_4_ molecules, likely due to competitive adsorption or changes in gas behavior under pressure.

Furthermore, the plots examining the relationship between CO_2_ adsorption and other variables, such as TOC, maturity, and temperature, show no significant trends or patterns. This lack of clear correlations suggests that these factors may not have a direct impact on the CO_2_ adsorption capacity of shale samples within the studied range. Similarly, the plots analyzing the relationship between CH_4_ adsorption and TOC, maturity, and temperature also show no discernible trends, indicating that these factors do not play a dominant role in determining methane adsorption capacity in shale samples.

Numerical correlation matrices are essential tools in ML and data analysis. These matrices represent the linear relationships between different variables and can be valuable in various processes such as feature selection, dimensionality reduction, and exploratory data analysis (EDA). In this study, the Pearson correlation coefficient is used to compute the thermal numerical correlation matrix shown in Fig. 3. The Pearson correlation coefficient is a statistical measure that quantifies the strength and direction of the linear relationship between two variables. It is represented by a value between − 1 and 1.

Pearson correlation coefficient values can be positive, negative, or zero (indicating no correlation). A perfect positive correlation means that as the value of one variable increases, the other variable increases in proportion. A perfect negative correlation means that as the value of one variable increases, the other variable decreases in proportion. No correlation indicates that there is no linear relationship between the two variables.

According to Eq. 3, the Pearson correlation coefficient is expressed as follows:

$$r=\frac{\sum {(X}_{i}-\overline{X }){(Y}_{i}-\overline{Y })}{\sqrt{{({X}_{i}-\overline{X })}^{2}-{({Y}_{i}-\overline{Y })}^{2}}}$$

In this equation,  ${X}_{i}$ and ${Y}_{i}$ represent the observed values, and $\overline{X }$ and $\overline{Y }$ are the mean values of variables $X$ and $Y$, respectively. Therefore, if $r>0$, a positive (direct) correlation exists, it should be noted that the closer the value of r is to 1, the stronger the positive relationship. Similarly, if $r<0$, a negative (inverse) correlation exists, and it should be noted that the closer the value of r is to − 1, the stronger the negative relationship. It is important to note that if $r=0$, no linear relationship exists, and the relationship may be nonlinear (in which case, no correlation is present).

In this study, the Pearson correlation coefficient and heatmap were employed to assess the relationships between various variables, such as temperature, pressure, rock type (shale and coal), TOC, moisture content, and the percentage of CO_2_ in the injected gas. This information can aid in process optimization and more effective decision-making.

The heatmap provides a comprehensive representation of the Pearson correlation coefficients between different parameters and the CO_2_ and CH_4_ adsorption capacities in shale samples. The intensity and color direction (red for positive correlation, blue for negative correlation) indicate the strength and direction of the linear relationship between each pair of variables.

One prominent trend observed in the heatmap is the strong positive correlation between CO_2_ percentage and CO_2_ adsorption capacity (0.58), suggesting that as the CO_2_ content in the shale gas mixture increases, the shale’s capacity to adsorb CO_2_ also rises. This relationship indicates that CO_2_ adsorption in shale is influenced by the partial pressure of CO_2_ in the gas phase, with higher CO_2_ concentrations leading to increased adsorption. Conversely, a notable negative correlation between CH_4_ adsorption and CO_2_ adsorption (− 0.16) suggests that the presence of CO_2_ may interfere with CH_4_ adsorption. This negative correlation could be due to competitive adsorption between CO_2_ and CH_4_ molecules for the same adsorption sites in the shale matrix.

Interestingly, the heatmap also reveals a strong positive correlation between CO_2_ percentage and TOC content (0.61), as well as between TOC and CO_2_ adsorption capacity (0.34). These correlations suggest that TOC plays a significant role in influencing CO_2_ adsorption in shale, possibly by providing additional adsorption sites or enhancing the overall adsorption capacity of the shale through its intrinsic physicochemical properties. In contrast, CH_4_ adsorption shows a weak correlation with TOC (− 0.10), indicating that TOC content may not be a major factor in influencing CH_4_ adsorption.

Additionally, the heatmap indicates a positive correlation between pressure and CO_2_ adsorption (0.18), suggesting that higher pressures facilitate CO_2_ adsorption. However, the correlation between pressure and CH_4_ adsorption is negative (− 0.17), implying that higher pressure may hinder CH_4_ adsorption. These opposing trends highlight the different behaviors of CO_2_ and CH_4_ under varying pressure conditions in the shale environment.

Furthermore, the heatmap shows a negative correlation between temperature and CO_2_ adsorption (− 0.11) and a positive correlation between temperature and CH_4_ adsorption (0.26). This suggests that temperature may affect the adsorption behavior of both gases, possibly through its effects on gas kinetics and the shale matrix’s characteristics.”

now reads

“The experimental dataset used in this study was compiled from the published work of Tavakolian et al.^1^. The dataset includes adsorption measurements of CH_4_ and CO_2_ in shale and coal reservoirs under a wide range of pressures, temperatures, and reservoir properties. Detailed descriptions of the experimental setup, data distribution, and statistical characteristics have been thoroughly reported in the original study and are therefore not repeated here. In the present work, the dataset is directly utilized as input for machine learning model development and comparative analysis.”

Additionally, in the Data collection and specific description section, under the subheading “Machine learning model”,

“In similar problems, ML models, particularly regression models, are utilized. These models help us better understand how changes in independent variables influence the dependent variable and how a relationship is established between them. Various learning methods are employed to define this relationship. In this study, five common methods that yield satisfactory results in such problems have been used. These methods include RF, CatBoost, AdaBoost, and ExtraTrees. Each of these methods is explained in detail below.”

now reads

“Due to the nonlinear and multivariate characteristics of gas sorption processes, as well as the heterogeneity of the experimental dataset, we chose ensemble-based machine learning techniques during this study for their capacity to effectively model complicated interactions between variables and provide less variance in comparison to single models when predicting outcomes from tabular experimental datasets.

Random Forest is a well-known ensemble algorithm that is based on bootstrap aggregation, retains strong performance, and is robust to overfitting when modeling nonlinear relationships. The Extra Trees ensemble method has been selected due to its similarity to the Random Forest method with the introduction of additional randomness when constructing trees. The additional randomization of the Extra Trees method will allow for an evaluation of whether the increased amount of randomness will improve generalization for the dataset in question.

AdaBoost is an example of a boosting type of ensemble technique that focuses on improving prediction accuracy through iterative treatment of samples that are difficult to predict based on their resampling through adaptive reweighting of instances. CatBoost is an example of a more recent variation of the Gradient Boosting Algorithm that performs particularly well with respect to complex nonlinear relationships and the structured nature of tabular format datasets.

Choosing these models allows for a standardized comparison of different ensemble learning techniques like bagging, randomization-based ensembles, and boosting techniques, all within a unified modeling framework. Simpler linear regression models were not considered, as they are generally insufficient to represent the highly nonlinear adsorption behavior observed in experimental adsorption datasets.”

Furthermore, in the Results and discussion section, under the subheading “Sensitivity analysis”,

“In general, sensitivity analysis is an effective tool for gaining a deeper understanding of the impact of various variables on gas adsorption. This method not only aids in improving modelling accuracy but also provides useful information for designing future experiments and optimizing operational conditions in gas adsorption systems.”

now reads

“In general, sensitivity analysis is an effective tool for gaining a deeper understanding of the impact of various variables on gas adsorption. This method not only aids in improving modeling accuracy but also provides useful information for designing future experiments and optimizing operational conditions in gas adsorption systems.

Unlike recent studies such as Tavakolian et al.^1^, which evaluated a broad range of machine learning techniques for modeling CH_4_ and CO_2_ sorption capacity and identified Random Forest as the most accurate predictor, the present study introduces a unified and systematic benchmarking framework for multiple ensemble-based algorithms, including Random Forest, CatBoost, AdaBoost, and Extra Trees. All models are implemented using identical data partitioning, preprocessing, and evaluation protocols, with Bayesian hyperparameter optimization consistently applied across the entire modeling pipeline. This standardized framework minimizes biases associated with unequal tuning strategies and enables a fair comparison of intrinsic model capabilities.

Beyond predictive accuracy, this study places stronger emphasis on interpretability and physical consistency by employing SHAP-based sensitivity analysis to quantify the influence of key parameters on CO_2_ and CH_4_ adsorption. While the feature importance trends reported by Tavakolian et al.^1^ are generally confirmed—particularly the dominant roles of pressure and TOC—the present analysis provides deeper insights into competitive adsorption mechanisms, revealing that pressure and CO_2_ concentration enhance CO_2_ adsorption while exerting an inverse effect on CH_4_ due to site competition. Moreover, although Extra Trees achieves lower MAE and MAPE values in certain cases, the CatBoost model demonstrates superior generalization performance and stability on unseen data, achieving R^2^ values of 0.9989 for CO_2_ and 0.9965 for CH_4_. These findings highlight important trade-offs between accuracy and robustness and offer practical guidance for selecting reliable machine learning tools for gas adsorption modeling in heterogeneous tight reservoirs.”

Finally, Figs. 1, 2, 3, Tables 2 and 3, and Eqs. 1–3 have been removed. The original Figures, Tables, and Equations with accompanying captions appear below. As a result of the changes, Figures, Tables, and Equations have been renumbered.

**Fig. 1:**
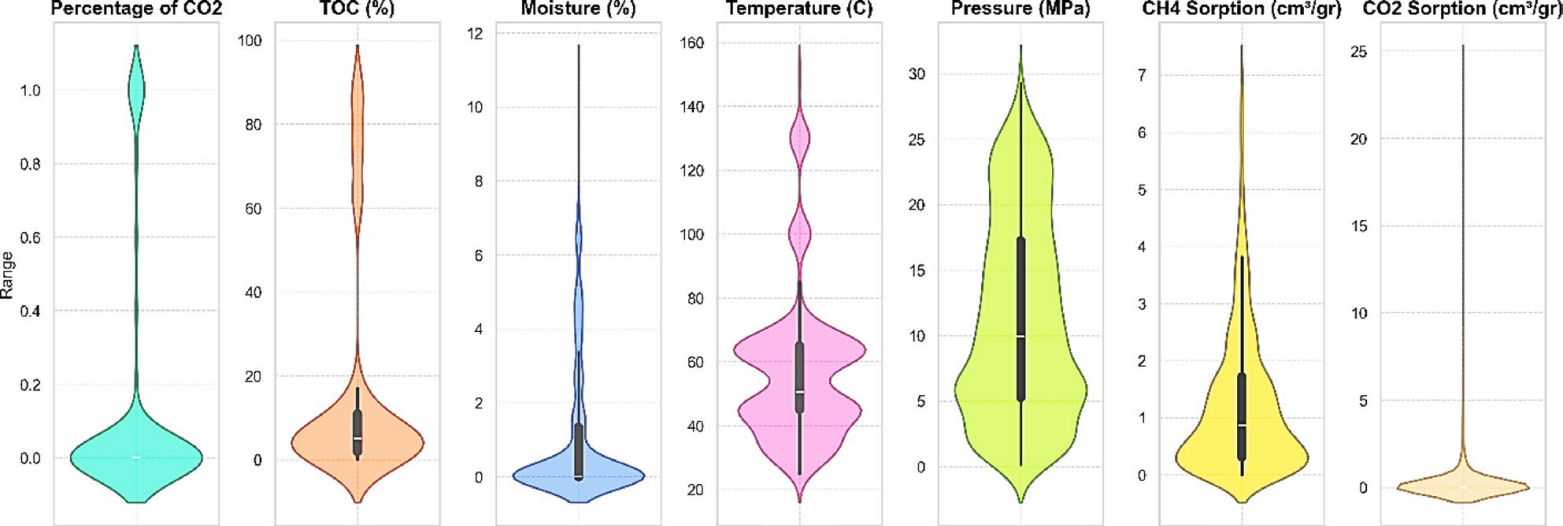
The violin plot for the examined data.

**Fig. 2:**
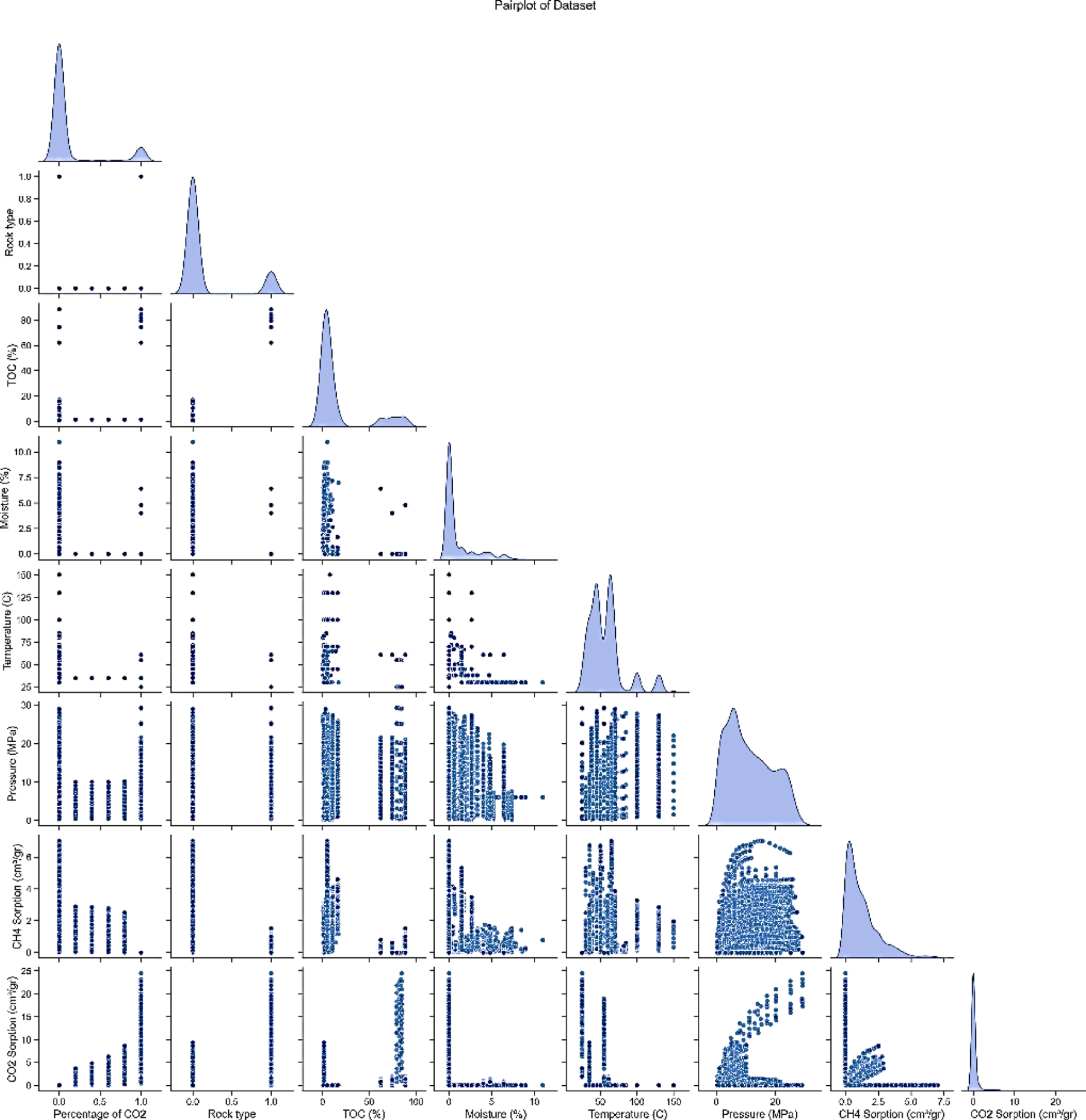
Pairwise plots related to methane and CO2 adsorption.

**Fig. 3:**
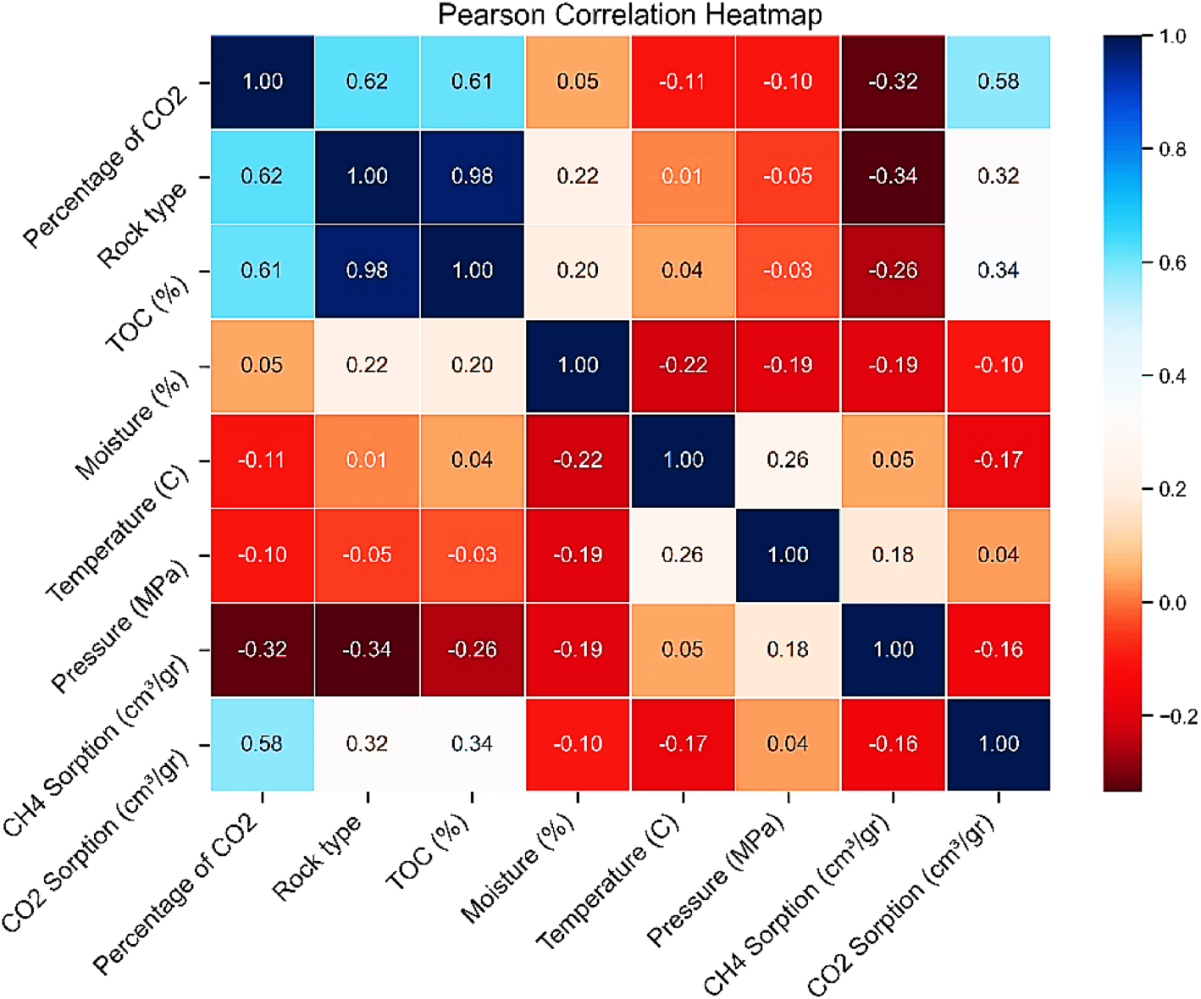
Heat map (Pearson correlation matrix).

**Table 2:** Statistical data.

	Percentage of CO_2_	TOC (%)	Moisture (%)	Temperature (C)	Pressure (MPa)	CH_4_ Excess Sorption (cm^3^/gr)	CO_2_ Excess Sorption (cm^3^/gr)
Max	1	88.500	10.9700	150.00	29.2473	7.0362	24.4681
Min	0	0.0900	0	25.00	0.1640	0	0
Range	1	88.4100	10.9700	125.00	29.0833	7.0362	24.4681
median	0	5.1500	0	50.400	9.9401	0.8651	0
Mod	0	5.4100	0	45.00	6.00	0	0
Mean	0.1228	16.2977	0.9741	57.04033	11.3166	1.2406	0.5303
Skewness	2.2939	1.8647	1.9610	1.5747	0.4080	1.6303	6.2580
Variance	0.0995	712.020	3.2584	556.4257	57.2000	1.5931	5.1165
Kurtosis	3.4193	1.7489	2.9405	2.7252	-0.9911	2.9989	44.1605

**Table 3:** Statistical summary of the available data using violin plots, pair plots, and heat maps.

Feature/relationship	Details and results
CO_2_ Percentage	Data is mostly concentrated at lower percentages, with some spread in higher ranges. This indicates that most samples have low CO_2_ concentrations, but a few outliers suggest variability in the gas composition among different samples.
TOC Percentage	TOC values are predominantly below 5%, reflecting the natural heterogeneity of organic matter in shale samples. Higher TOC levels could potentially influence adsorption capacity due to their impact on microporosity and adsorption sites.
Moisture	The distribution of moisture shows a wide range with significant variability. Samples with higher moisture content might have reduced gas adsorption due to competitive water adsorption at adsorption sites.
Temperature	Temperatures range from 20 to 160°C, covering a wide spectrum of conditions. This broad range reflects the varying geothermal gradients and reservoir conditions, which significantly influence gas adsorption and desorption behaviors.
Pressure	Pressure data is mainly concentrated above 10 MPa, highlighting the high-pressure conditions typical of gas storage in shale reservoirs. Such pressures are critical for assessing the adsorption and phase behavior of gases under reservoir-like conditions.
CH_4_ and CO_2_ Adsorption	Adsorption values for both gases are generally low, indicating limited adsorption capacity in some shale samples, possibly due to low TOC or less-developed pore structures.
CO_2_ Adsorption and Pressure	A clear positive trend; as pressure increases, CO_2_ adsorption rises. This indicates that pressure is a key driver in enhancing CO_2_ storage capacity in shale by increasing gas density and facilitating gas adsorption within nanopores.
CH_4_ Adsorption and Pressure	Negative trend; higher pressure reduces CH_4_ adsorption. This may result from competitive adsorption with CO_2_ or changes in gas phase behavior at elevated pressures, leading to preferential adsorption of CO_2_ over CH_4_.
CO_2_ Percentage and CO_2_ Adsorption	Strong positive correlation (0.58) suggests that higher CO_2_ concentrations in the injected gas significantly enhance CO_2_ adsorption. This relationship emphasizes the role of partial pressure in determining adsorption efficiency.
CH_4_ Adsorption and CO_2_ Adsorption	Noticeable negative correlation (-0.16) indicates competitive adsorption between CO_2_ and CH_4_. As CO_2_ adsorption increases, CH_4_ adsorption decreases, likely due to competition for limited adsorption sites.
CO_2_ Percentage and TOC	Strong positive correlation (0.61) highlights the role of TOC in influencing gas composition and its interaction with shale, potentially by providing additional microporous sites for CO_2_ adsorption.
TOC and CO_2_ Adsorption	Moderate positive correlation (0.34) indicates that TOC enhances CO_2_ adsorption. This is likely due to the presence of organic matter with higher affinity for CO_2_, increasing the overall adsorption capacity.
TOC and CH_4_ Adsorption	Weak negative correlation (-0.10) suggests TOC has a negligible or slightly adverse effect on CH_4_ adsorption. This might result from differences in the molecular interaction of CH_4_ and CO_2_ with organic matter.
Pressure and CO_2_ Adsorption	Weak positive correlation (0.18) shows that higher pressure moderately facilitates CO_2_ adsorption. This aligns with the observed density-dependent adsorption behavior of CO_2_ in shale reservoirs.
Pressure and CH_4_ Adsorption	Weak negative correlation (-0.17) indicates that increasing pressure may slightly hinder CH_4_ adsorption, possibly due to the dominance of CO_2_ at higher pressures.
Temperature and CO_2_ Adsorption	Weak negative correlation (-0.11) suggests that higher temperatures might reduce CO_2_ adsorption, likely due to increased gas desorption rates and reduced adsorption affinity at elevated temperatures.
Temperature and CH_4_ Adsorption	Moderate positive correlation (0.26) indicates that CH_4_ adsorption may slightly increase with temperature, possibly due to changes in gas mobility and shale properties, though the effect is not strong.

The original Article has been corrected.

